# Prevalence of *Spiroplasma* and interaction with wild *Glossina tachinoides* microbiota[Fn FN1]

**DOI:** 10.1051/parasite/2023064

**Published:** 2023-12-19

**Authors:** Kiswend-Sida M Dera, Mouhamadou M Dieng, Percy Moyaba, Gisele MS Ouedraogo, Soumaïla Pagabeleguem, Flobert Njokou, François S Ngambia Freitas, Chantel J de Beer, Robert L Mach, Marc JB Vreysen, Adly MM Abd-Alla

**Affiliations:** 1 Insect Pest Control Laboratory, Joint FAO/IAEA Centre of Nuclear Techniques in Food and Agriculture 1400 Vienna Austria; 2 Insectarium de Bobo Dioulasso – Campagne d’Eradication de la mouche tsetse et de la Trypanosomose (IBD-CETT) 01 BP 1087 Bobo Dioulasso 01 Burkina Faso; 3 Université Gaston Berger Saint Louis Senegal; 4 Epidemiology, Parasites and Vectors, Agricultural Research Council-Onderstepoort Veterinary Research (ARC-OVR) Pretoria South Africa; 5 University of Dedougou B.P. 176 Dédougou 01 Burkina Faso; 6 Laboratory of Parasitology and Ecology, Faculty of Sciences, University of Yaounde I Po. Box 812 Yaoundé Cameroon; 7 Centre for Research in Infectious Diseases (CRID) Po. Box 13591 Yaoundé Cameroon; 8 Institute of Chemical, Environmental, and Bioscience Engineering, Research Area Biochemical Technology, Vienna University of Technology, Gumpendorfer Straße 1a 1060 Vienna Austria

**Keywords:** *Trypanosoma* spp., Microbe infection rate, Interactions, Paratransgenesis

## Abstract

Tsetse flies (Diptera: Glossinidae) are vectors of the tropical neglected diseases sleeping sickness in humans and nagana in animals. The elimination of these diseases is linked to control of the vector. The sterile insect technique (SIT) is an environment-friendly method that has been shown to be effective when applied in an area-wide integrated pest management approach. However, as irradiated males conserve their vectorial competence, there is the potential risk of trypanosome transmission with their release in the field. Analyzing the interaction between the tsetse fly and its microbiota, and between different microbiota and the trypanosome, might provide important information to enhance the fly’s resistance to trypanosome infection. This study on the prevalence of *Spiroplasma* in wild populations of seven tsetse species from East, West, Central and Southern Africa showed that *Spiroplasma* is present only in *Glossina fuscipes fuscipes* and *Glossina tachinoides*. In *G. tachinoides,* a significant deviation from independence in co-infection with *Spiroplasma* and *Trypanosoma* spp. was observed. Moreover, *Spiroplasma* infections seem to significantly reduce the density of the trypanosomes, suggesting that *Spiroplasma* might enhance tsetse fly’s refractoriness to the trypanosome infections. This finding might be useful to reduce risks associated with the release of sterile males during SIT implementation in trypanosome endemic areas.

## Abstract (fr)

Les mouches tsé-tsé (Diptera : Glossinidae) sont les vecteurs de maladies tropicales négligées, la maladie du sommeil chez l’homme et la nagana chez les animaux. L’élimination de ces maladies est liée à la lutte contre le vecteur. La technique de l’insecte stérile (TIS) est une méthode respectueuse de l’environnement qui s’est révélée efficace lorsqu’elle est appliquée dans le cadre d’une approche de lutte antiparasitaire intégrée à l’échelle d’une zone. Cependant, comme les mâles irradiés conservent leur compétence vectorielle, il existe un risque potentiel de transmission des trypanosomes lors de la libération des mâles sur le terrain. L’analyse de l’interaction entre la mouche tsé-tsé et son microbiote, et entre différents microbiotes et le trypanosome, pourrait fournir des informations importantes pour améliorer la résistance de la mouche à l’infection trypanosomienne. Cette étude sur la prévalence de *Spiroplasma* dans les populations sauvages de sept espèces de glossines d’Afrique de l’Est, de l’Ouest, centrale et australe a montré que *Spiroplasma* est présent uniquement chez *Glossina fuscipes fuscipes* et *Glossina tachinoides*. Chez *G. tachinoides*, un écart significatif par rapport à l’indépendance dans la co-infection par *Spiroplasma* et *Trypanosoma* spp. a été observé. De plus, les infections à *Spiroplasma* semblent réduire considérablement la densité des trypanosomes, ce qui suggère que *Spiroplasma* pourrait renforcer le caractère réfractaire de la mouche tsé-tsé aux infections trypanosomiennes. Cette découverte pourrait être utile pour réduire le risque associé à la libération de mâles stériles lors de la mise en œuvre de la TIS dans les zones d’endémie trypanosomienne.

## Introduction

Tsetse flies (*Diptera*: *Glossinidae*) transmit trypanosomes, the causative agent of one of the most neglected vector-borne diseases in sub-Saharan Africa, *i.e.*, African animal trypanosomosis or AAT (also called nagana) and human African trypanosomosis or HAT (sleeping sickness) [9, 37]. Tsetse flies are principally hematophagous and feed exclusively on vertebrate blood [2, 57]. During a blood meal on an infected host, the fly can ingest the trypanosomes which are established in the midgut. After several series of proliferation and differentiation, the trypanosomes mature in the salivary gland or the mouth parts depending on the trypanosome species. The parasite can then be transmitted to a mammalian host during a subsequent blood meal [60, 61].

The lack of effective prophylactic drugs or a vaccine [9], and the development of resistance to trypanocidal drugs [17], makes tsetse control the most efficient alternative for sustainable management of these diseases. One effective method for tsetse control is the Sterile Insect Technique (SIT) that needs to be implemented as part of an area-wide integrated pest management (AW-IPM) approach. The sterile insect technique requires mass production of the target insect, sterilization with irradiation and the release of these sterile insects in the field to mate with wild females to reduce fertility of the targeted population. However, the irradiation does not affect the tsetse fly’s susceptibility to develop mature trypanosome infections [18], and hence the desirability to enhance refractoriness of tsetse flies for trypanosome infections that would be used for release in an SIT program [59].

Symbiotic associations have been described in insects and typically involve bacteria that are vertically transmitted through progeny and may influence several functions of their hosts [51]. Tsetse flies harbor tree major endosymbiotic bacteria, *i.e.*, the obligate mutualist *Wigglesworthia glossinidia*, the mutualist *Sodalis glossinidius*, and the parasitic *Wolbachia pipientis* [42]. Recently, a fourth endosymbiont, *i.e.*, *Spiroplasma* was discovered in some natural tsetse populations and laboratory colonies of *Glossina palpalis palpalis, Glossina fuscipes fuscipes*, and *Glossina tachinoides,* all three being members of the palpalis group [21, 28]. In addition, multilocus sequencing typing (MLST) analysis identified two different strains of *Spiroplasma* in *G. f. fuscipes* and *G. tachinoides* [21].

Bacteria belonging to the genus *Spiroplasma* are Gram-positive, wall-less and are described in arthropods and plants. They are classified into three major monophyletic groups based on the 16S ribosomal RNA gene (rDNA) sequence: Ixodetis, Citri-Chrysopicola-Mirum (CCM), and Apis [24, 27]. They belong to the class of Mollicutes and are characterized by an helical shape and the lack of a cell wall and are only enveloped by a cholesterol-containing cell membrane [26]. The *Spiroplasma* are unique in having a well-defined, dynamic, helical cell geometry and a flat, monolayered, membrane-bound cytoskeleton, which follows, intracellularly, the shortest helical line on the cellular coil. They have a cytoskeleton which controls both the dynamic helical shape and the consequent motility of the cell [47, 58]. Their cell size varies between 100 and 240 nm [1]. The genome size ranges from 780 to 2,220 kb and is rich in AT [1, 47]. The role of *Spiroplasma* in the tsetse fly host is currently unclear, but it has been reported to have a negative effect on the viability of *Harmonia axyridis*, and male killing activity in *Drosophila melanogaster* and *Drosophila neotestacea* [5, 24]. Many studies have also revealed that *Spiroplasma* might cause disease in arthropods and plants [5, 39]. Conversely, some *Spiroplasma* strains might have a positive effect in their hosts conferring resistance against pathogens [34, 41, 46]. Like *Wolbachia*, *Spiroplasma* can be found in ovaries. However, they primarily reside in the hemolymph but can also be detected in fat body and salivary glands. Using a laboratory colony of *G. f. fuscipes*, it was shown that *Spiroplasma* may interact with trypanosomes [50]. Flies harboring *Spiroplasma* presented a lower prevalence of trypanosome infection in the midgut, indicating a potential negative correlation between *Spiroplasma* presence and trypanosome infection. In the same study, it was shown that *Spiroplasma* can be transmitted vertically, although the possibility of horizontal transmission could not be excluded. These findings supported the use of *Spiroplasma* in paratransgenesis approaches to develop trypanosome refractoriness. The use of paratransgenesis has been suggested as an approach that could confer resistance against pathogens by genetically engineering the symbionts or the vector [7, 14]. This approach has been implemented successfully in triatome bugs [22] and mosquitoes [62], but is still under evaluation for tsetse flies. In this respect, the use of the endosymbiont *Sodalis* was previously recommended for paratransgenic approaches in tsetse flies [6, 15, 16]. In support of this idea, irradiating 22 day-old pupae did not impact the copy number of *Sodalis* in *G. morsitans morsitans* compared to non-irradiated flies [18]. However, it has been reported that *Sodalis* has a negative impact on the metabolic and reproductive fitness of *G. f. fuscipes* [56].

In this study, the prevalence of *Spiroplasma* infection was assessed in natural tsetse populations collected from different countries in Africa. The potential interaction between *Spiroplasma* with the trypanosomes and *Wigglesworthia* was studied using a *G. tachinoides* population from Burkina Faso and Ghana. We also report on the genotyping and presence of different *Spiroplasma* strains in wild *G. tachinoides* populations.

## Materials and methods

### Tsetse taxon collection and DNA purification

**Figure 1:**
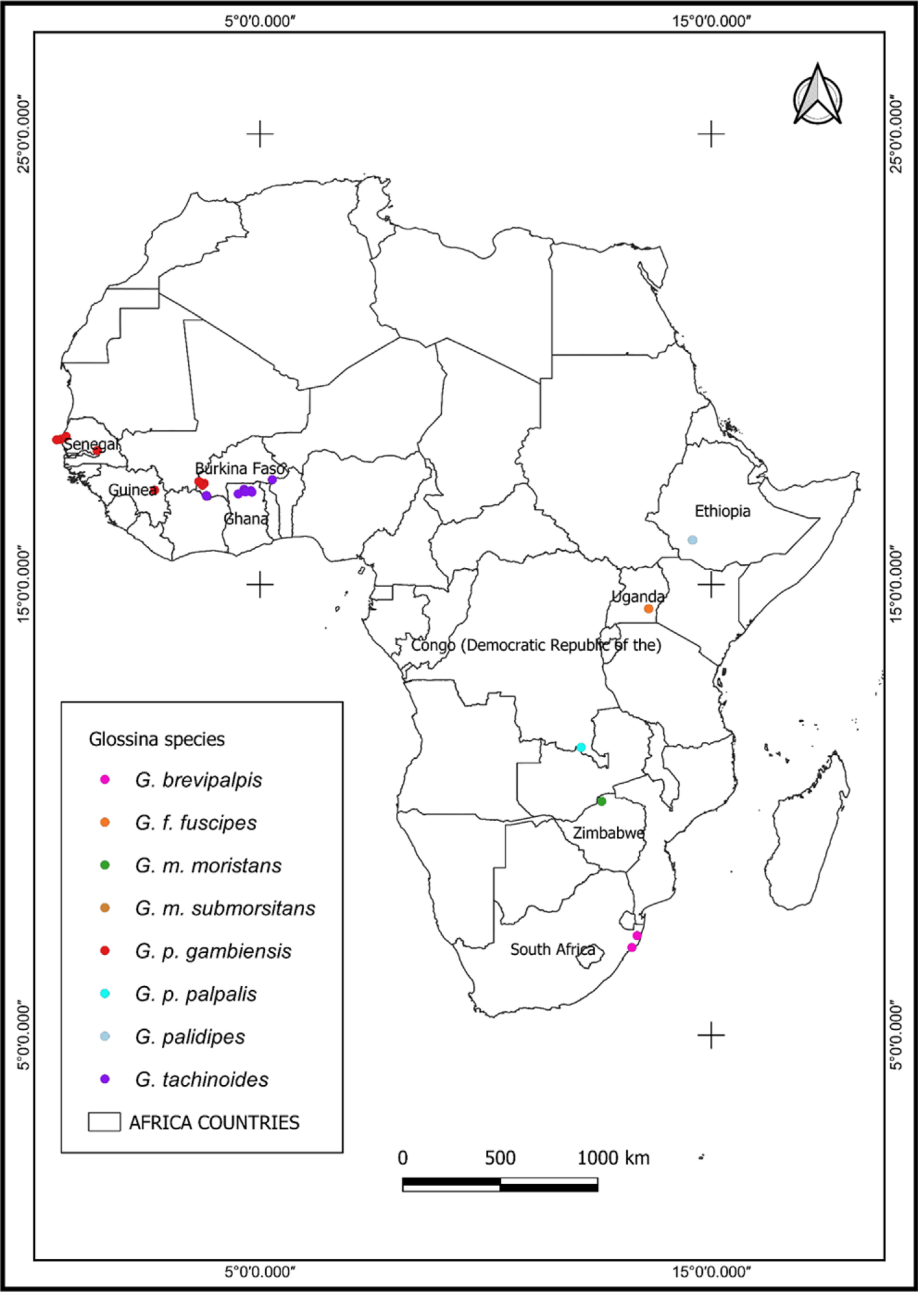
Geographical locations of tsetse samples in Africa.

Wild populations of tsetse flies were collected in 40 locations in 10 different countries in West Africa (Burkina Faso, Ghana, Guinea, Mali and Senegal), Central Africa (Democratic Republic of Congo), East Africa (Ethiopia and Uganda) and southern Africa (South Africa and Zimbabwe). Eight tsetse taxa were analysed, including *G. brevipalpis, G. f. fuscipes, G. m. morsitans, Glossina morsitans submorsitans, Glossina pallidipes, Glossina palpalis gambiensis, Glossina palpalis palpalis* and *G. tachinoides* (Fig. 1, Supplementary Table 1).

Adult flies were collected in 1995 and between 2005 and 2018 using the biconical Challier-Laveissière trap, the monoconical Vavoua trap [11, 13], the Ngu trap, the Epsilon trap baited with acetone [25], and the odour baited H trap [32]. The collected flies were stored in 95% absolute ethanol or propylene glycol and shipped to the Insect Pest Control Laboratory (IPCL) of the Joint FAO/IAEA Centre of Nuclear Techniques in Food and Agriculture, Seibersdorf, Austria. At the IPCL, the samples were stored at −20 °C until further use. Total DNA was extracted from the whole body of each individual fly using a DNeasy tissue kit (QIAGEN Inc., Valencia, CA, USA), following the supplier’s instructions.

### Prevalence of *Spiroplasma* and *Trypanosoma*

To detect *Spiroplasma* infection, PCR amplification of an approximately 455 bp fragment of the 16S *rRNA* gene was performed [21]. The PCR was carried out in 25 μL reaction mixtures containing 22.5 μL of 1.1× Pre-Aliquoted PCR Master Mix (0.625 units Thermoprime Plus DNA Polymerase, 75 mM Tris–HCl (pH 8.8 at 25 °C), 20 mM (NH_4_)2SO4, 2.0 mM MgCl_2_, 0.01% (v/v) Tween-20 and 0.2 mM each of the dNTPs (ABgene, UK), and 1.5 μL of template DNA plus 1 μL of *Spiroplasma* 16S RNA primers (63F and TKSS) (Supplementary Table 2) to a final concentration of 0.2 mM per primer. PCR conditions were 95 °C for 5 min, followed by 34 cycles of 95 °C for 30 s, 58 °C for 30 s, 72 °C for 30 s, and final extension 72 °C for 10 min. PCR products were electrophoresed in 2% molecular grade agarose (Fisher Biotech) stained with SafeGreen. DNA from the IPCL colony of *G. f. fuscipes*, which is known to be infected with *Spiroplasma*, and sterilized distilled water were included in each PCR test as positive and negative controls, respectively. As described previously [19], the *Glossina* species microsatellite GpCAG133 was used to control the quality of the extracted DNA and only validated samples were considered for *Spiroplasma* or *Trypanosoma* infection status. To confirm that the amplified PCR products obtained with *G. pallidipes, G. m. morsitans,* and *G. p. gambiensis* were *Spiroplasma-*specific sequences, two approaches were used: the first was to confirm the specificity of the amplification by performing PCR using the MLST primers shown in Supplementary Table 2. The second was to sequence the PCR product obtained by the 16S *rRNA* gene primers. For the sequencing, PCR products were purified using the High Pure PCR Clean-up Micro Kit (Roche, Basel, Switzerland) and ligated to the pGEM-T vector (Promega, Madison, WI, USA), following the supplier’s instructions. The recombinant plasmids were transformed into DH5α-competent bacteria (Invitrogen, Carlsbad, CA, USA), following the supplier’s instructions. The recombinant plasmids and the inserted sequences were confirmed by Sanger sequencing (Eurofins Genomics, Ebersberg, Germany) with the universal vector primers M13F_uni (-21) (5′–TGT AAA ACG GCC AGT–3′) and M13R_rev (-29) (5′–CAG GAA ACA GCT ATG ACC–3′). For the other tsetse species including *G. f. fuscipes, G. brevipalpis* and *G. tachinoides*, the amplified PCR products were purified with the ZR-96 DNA Clean & Concentrator^®^-5 (Zymo Research, Irvine, CA, USA), following the manufacturer’s protocol and submitted directly to sequencing without cloning using the 63F and TKSS primers (Eurofins Genomics, Ebersberg, Germany). The resulting sequences were blasted against the non-redundant protein sequence (nr) database in the NCBI server using the BLAST tool https://blast.ncbi.nlm.nih.gov/Blast.cgi to identify and annotate the sequence. The sequence was considered a *Spiroplasma* sequence if it matched with *Spiroplasma* sequence in the database. The prevalence of *Trypanosoma* was assessed as in Ouedraogo *et al.* [43].

### Analysis of the interaction between *Spiroplasma* and *Trypanosoma* in *G. tachinoides*

Co-infection of *Spiroplasma* and *Trypanosoma* spp. was evaluated by PCR. Trypanosome prevalence was determined as previously described [43]. Infection status was divided into four categories: 1. *Spiroplasma* positive and *Trypanosoma* positive (Sp^+^/T^+^), 2. *Spiroplasma* positive and *Trypanosoma* negative (Sp^+^/T^−^), 3. *Spiroplasma* negative and *Trypanosoma* positive (Sp^−^/T^+^), and 4. *Spiroplasma* negative and *Trypanosoma* negative (Sp^−^/T^−^).

### Analysis of the density of *Spiroplasma, Trypanosoma* and *Wigglesworthia* infection density

Samples showing the following infection status (Sp^+^/T^+^), (Sp^+^/T^−^), and (Sp^+^/T^−^) were used to assess the density of *Spiroplasma, Trypanosoma* spp., and *Wigglesworthia* using relative quantitative PCR (qPCR). The qPCR was performed using a CFX96 Real Time PCR Detection System (Bio-Rad, Hercules, CA, USA). The *Spiroplasma* density was assessed by the amplification of 16S *rRNA* gene with the qPCR *Spiroplasma* primers (Supplementary Table 2). In addition, the density of *Wigglesworthia* was evaluated as previously described [18] using the *thiC* (thiamine biosynthesis gene) (Supplementary Table 2). Based on the above-mentioned criteria, 212 *G. tachinoides* individuals (76, 65 and 71 flies with (Sp^+^/T^+^), (Sp^+^/T^−^), and (Sp^−^/T^+^) infection status, respectively) were selected from Burkina Faso and Ghana samples*.* In addition, samples with (Sp^+^/Tryp^+^), (Sp^+^/T^−^), and (Sp^−^/T^+^) were used to assess the impact of *Spiroplasma* infection on *Trypanosoma* density. Trypanosomatidae 18S *rRNA* gene specific primers (18S_Typ_F and 18S_Typ_R) (Supplementary Table 2) were used to assess the *Trypanosoma* density in the tested samples. The DNA from all selected samples was diluted to a final concentration of 4 ng/μL and 5 μL of the diluted DNA was used for qPCR to determine *Spiroplasma*, *Wigglesworthia*, and *Trypanosoma* DNA density normalized to the housekeeping *β-tubulin* gene. The amplification mixture contained 5 μL of DNA template, 200 nM of each primer, and 7.5 μL iQ^TM^ SYBER Green Supermix (Bio-Rad). qPCR cycling conditions for *Spiroplasma* and *Wigglesworthia* were as follows: initial denaturation at 95 °C for 2 min; 39 cycles of 95 °C for 5 s, 55 °C for 30 s, one step at 95 °C for 5 s, and a melting curve constructed from 65 °C to 95 °C in increments of 0.5 °C for 5 s. The same conditions were used for *Trypanosoma*, except the annealing temperature, which was 60 °C. Analysis of the *Spiroplasma, Wigglesworthia, Trypanosoma*, and *β-tubulin* densities was based only on the qPCR data with the expected melting curve at 81.5 °C, 85.5 °C, and 86 °C, respectively.

### Genetic variation and phylogenetic analysis of *Spiroplasma* in *G. tachinoides*

To assess the genetic variation of *Spiroplasma* in wild *G. tachinoides*, an MLST approach was employed on positive samples from Burkina Faso and Ghana using the following genes: 16S *rRNA*, *Spiroplasma* fructose repressor (*fruR*), *Spiroplasma* DNA Topoisomerase 4 subunit B (*parE*), and RNA polymerase subunit beta (*rpoB*). Primer sets used for each reaction, product sizes, and PCR conditions are shown in Supplementary Table 2.

All amplified PCR products were purified using a High Pure PCR Cleanup Micro Kit (Roche Diagnostics, Indianapolis, IN, USA) and a ZR-96 DNA Clean-up Kit™ (Zymo Research, Irvine, CA, USA). Sequencing was performed with Eurofins Genomics Company (https://www.eurofinsgenomics.com) and sequencing data were first analyzed using Geneious Prime^®^ 2023.0.2 and then blasted using the “Blast” resource of NCBI to confirm them as *Spiroplasma*. Phylogenetic trees were built for each gene (*16S rRNA, fruR, pare*, and *rpoB*) and for the concatenated data set using all four gene sequences. Multiple alignments were then performed using MUSCLE alignment with the default parameters on Geneious Prime^®^ 2023.0.2 and the Neighbor-joining tree was built using the Tamura-Nei genetic distance model.

### Data analysis

Prevalence data for *Spiroplasma* and *Trypanosoma* spp. were analysed in R using Rstudio version V 1.4.1106 [4] and general linear model (glm) [45] combined with analysis of variance, respectively provided by the ggplot package [63] to detect potential differences between countries and between localities in each country. *Spiroplasma*, *Trypanosoma*, and *Wigglesworthia* density levels were first normalized with the tsetse house-keeping *β-tubulin* gene and only the samples for which density levels were available for all three microorganisms were used for further analysis. The analysis was performed with the glm to detect the significant differences between the density of *Spiroplasma*, *Trypanosoma*, and *Wigglesworthia* according to the *Spiroplasma* and *Trypanosoma* co-infection status (Supplementary File 1). To evaluate the association between *Spiroplasma* and *Trypanosoma*, the Cochran-Manthel-Haenzel (CMH) test and the chi-square test were performed on the excel table, as described previously [19].

## Results

### Prevalence of *Spiroplasma*

The presence of *Spiroplasma* in wild populations of tsetse flies was assessed using a PCR-based method to amplify part of the 16S *rRNA* gene. Positive samples were identified based on the observed amplicon band size in the electrophoresis gel for all tsetse species. Sequencing of the respective PCR amplicons revealed that *Spiroplasma* infection was only confirmed in *G. tachinoides* (*N* = 41) and *G. f. fuscipes* (*N* = 6), both belonging to the *palpalis* subgenus (Table 1). In the case of *G. brevipalpis*, *G. m. morsitans*, *G. m. submorsitans, G. pallidipes, G. p. gambiensis*, and *G. p. palpalis*, the amplified sequence belonged to different microbial species, primarily *Bacillus cereus*, *Bacillus thuringiensis*, *Enterococcus cecorum*, and some uncultured bacteria (Data not shown).

**Table 1:** *Spiroplasma* identification in 8 *Glossina* species from 10 different countries using 16S *rRNA*, multilocus sequence typing (MLST), and Sanger sequencing.

Species	Country	Location	Total number of analyzed flies	Number of flies with valid DNA	Nb of *Spiroplasma*-positive using 16sRNA^a^	Samples positive with MLST/Samples tested with MLST^b^	Samples successfully sequenced^c^	Confirmation after sequencing
*G. brevipalpis*	South Africa	Zululand	50	0	0			
		**Colony**	**94**	**94**	**37**	**0/16**	**4**	No
		Phinda	180	180	0			
***G. fuscipes fuscipes***	**Uganda**	**Buvuma Island**	**147**	**94**	**6**	**6/6**	**6**	Yes
***G. morsitans morsitans***	**Zimbabwe**	**Makuti**	**94**	**94**	**17**	**0/6**	**4**	No
*G. morsitans submorsitans*	Burkina Faso	Singou	3	3	0			
		Comoe	32	31	4	0/4		
		**Folonzo**	**152**	**135**	**24**	**0/12**	**2**	No
*G. palpalis gambiensis*	Senegal	**Kedougou**	**62**	**60**	**57**	**0/22**	**4**	No
		Pout	207	199	169	0/18		
		Sebikotane	41	39	23	0/12		
		Diaka Madia	80	79	44	0/41		
		Diacksao Peulh	70	65	62			
		Hann	31	28	24	0/21		
	Guinea	Kansaba	32	31	29	0/9		
		Minipontda	32	29	15	0/6		
		Kindoya	87	83	80	0/66		
		Ghanda Oundou	27	20	14	0/10		
		Fefe	10	10	7	0/2		
		Togoue	21	21	21	0/21		
		Alahine	13	12	12	0/12		
		Boureya Kolonko	60	46	46	0/38		
	Mali	Fijira	14	14	11	0/11		
		Astan	138	126	85	0/32		
	Burkina Faso	Comoe	116	82	69	0/26		
		Kenedougou	12	12	5			
		Folonzo	153	123	70	0/23		
		Moussodougou	54	49	1			
		Kartasso	136	118	107	0/20		
***G. palpalis palpalis***	**Republic Democratic of Congo**	**Katanga**	**44**	**23**	**4**	**0/4**	**4**	No
***G. pallidipes***	**Ethiopia**	**Arba mich**	**94**	**94**	**24**	**0/8**	**8**	No
*G. tachinoides*	Burkina Faso	Comoe	119	119	29	24/24	24	Yes
		Folonzo	347	347	188	17		
		Colony (CIRDES)*	**25**	**19**	**2**	**2/2**	**2**	Yes
	Ghana	**Walewale**	**108**	**108**	**47**	**15/38**	**15**	Yes
		Mortani	41	41	40			
		Fumbissi	14	14	4	4/4		Yes
		Sissili Bridge	6	6	3			
		Grogro	11	11	4			
		Kumpole	7	7	1			

a First, Spiroplasma positive flies were identified using primers designed for the 16S *rRNA* gene sequence.

b Samples that were found to be positive then were tested using the multilocus sequence typing (MLST) gene to confirm the presence of *Spiroplasma*.

c *Spiroplasma-*positive samples confirmed by both primers (16S *rRNA*, MLST) or at least the 16S *rRNA* gene for each species were sequenced.

Samples sequences that did not match with the *Spiroplasma* sequences and matched with other bacteria were not considered positive for *Spiroplasma* infection.

* Samples from colony maintained in CIRDES.

Bold indicates successful sequencing.

**Figure 2:**
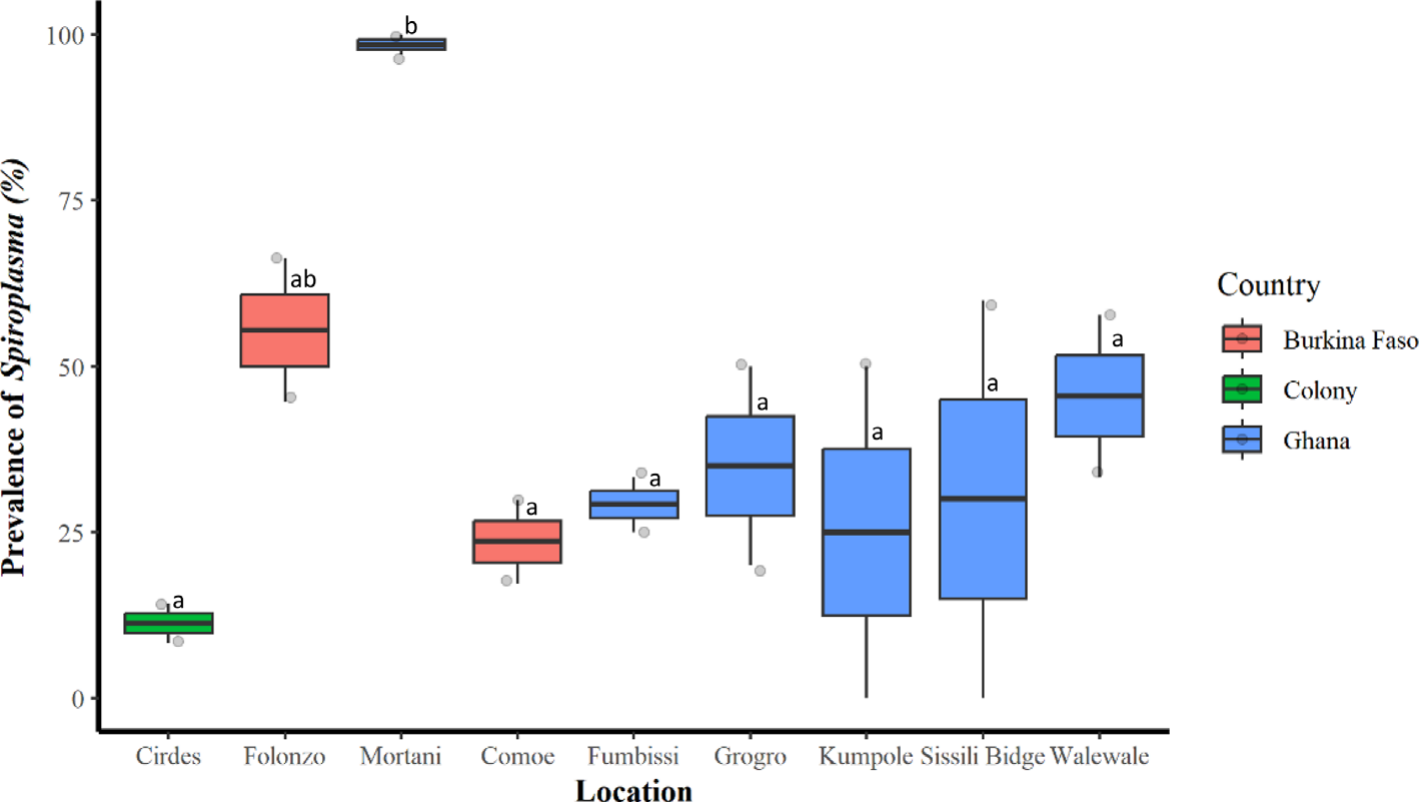
Prevalence of *Spiroplasma* according to location. Bars marked with the same lower-case letter do not differ significantly at the 0.05 level.

**Figure 3:**
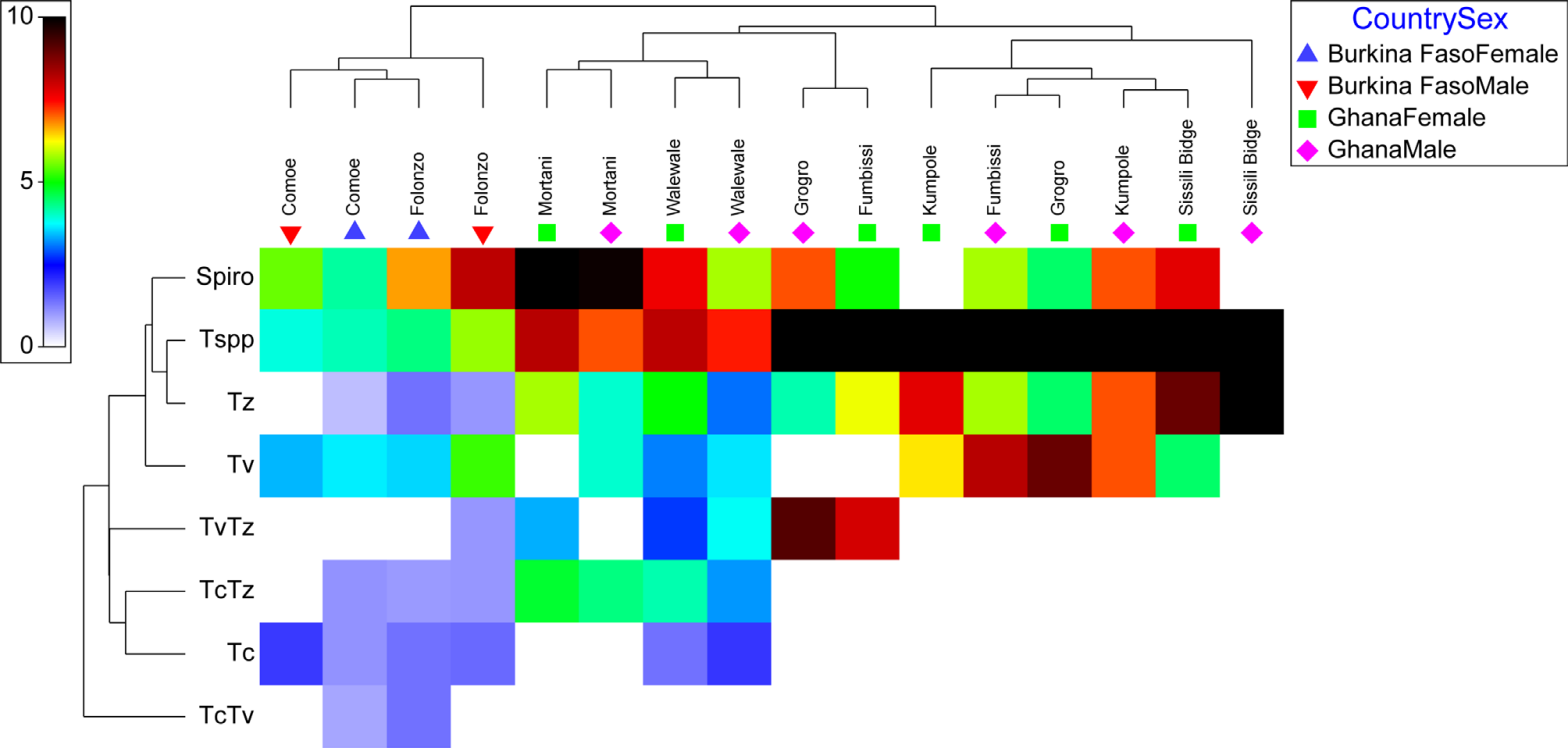
Prevalence of *Spiroplasma* and *Trypanosoma* (single and multiple) infections per country, location, and sex. Prevalence data were square root transformed and averaged based on location-sex and the matrix display was conducted in PRIMER version 7 + software. Tree on the left of the matrix is the similarity dendrogram based on the similarity index of the square root of the prevalence values. The color index is the square root of the prevalence values ranged 0–9 which is the square root of 0–81% prevalence.

The PCR results indicated an overall *Spiroplasma* prevalence of 39.27% in *G. tachinoides.* The prevalence did not differ significantly between Burkina Faso, Ghana, and the laboratory colony (χ^2^ = 2.12, df = 2, and *p* = 0.34), with Burkina Faso and Ghana showing a prevalence rate of 46.56% and 52.94%, respectively (Table 2). However, a significant variation in *Spiroplasma* prevalence was found across the various sampling locations (χ^2^ = 22.61, df = 8, and *p* = 0.003) (Table 2 and Figs. 2 and 3). Specifically, there was a significant difference in prevalence between the two sampling locations in Burkina Faso (χ^2^ = 6.459, df = 1, and *p* = 0.01), with a higher prevalence observed in Folonzo. Similarly, a significant difference was found between the prevalence rate in different locations in Ghana (χ^2^ = 11.955, df = 5, and *p* = 0.03), with the highest prevalence observed in the Mortani region (98.44%), where 100% of the female flies were infected. Conversely, the lowest prevalence of *Spiroplasma* was recorded in Kumpole, Ghana (25%), with male flies showing no sign of infection (Table 2, Figs. 2 and 3).

**Table 2:** Global prevalence of *Spiroplasma* in *Glossina tachinoides* according to locations and countries.

Country	Location	Sex	*Spiroplasma* prevalence (%)	*Trypanosoma* prevalence (%)*
Burkina Faso	Comoe	F	17.31	16.54
	Comoe	M	29.85	15
	Folonzo	F	44.56	18.87
	Folonzo	M	66.23	32.63
	Subtotal		46.56	20.76
Colony	CIRDES	F	14.29	–
	CIRDES	M	8.33	–
	Subtotal		11.31	–
Ghana	Walewale	F	57.78	66.04
	Walewale	M	33.33	53.85
	Sissili Bridge	F	60.00	100
	Sissili Bridge	M	0.00	100
	Fumbissi	F	25.00	100
	Fumbissi	M	33.33	100
	Kumpole	F	0.00	100
	Kumpole	M	50.00	100
	Grogro	F	20.00	100
	Grogro	M	50.00	100
	Mortani	F	100.00	66.67
	Mortani	M	96.88	50
	Subtotal		52.94	86.38
	Total (average)		39.27	69.97

* Data already published in Ouedraogo *et al.*, 2018 [43].

### Prevalence of single and multiple *Trypanosoma* infections

The screening of the flies indicated the presence of different taxa of *Trypanosoma*, including *Tc* (*Trypanosoma congolense type: Savanah, Kilifi, Forest*), *Tv* (*Trypanosoma vivax*), and *Tz* (*Trypanozoon* sp*.: Trypanosoma brucei brucei, Trypanosoma brucei gambiense, Trypanosoma brucei rhodesiense, Trypanosoma evansi*). The overall prevalence of single or multiple *Trypanosoma* infections among all tested flies was 69.97% (457/653). The prevalence of *Trypanosoma* varied significantly between countries (χ^2^ = 37.18, df = 1, and *p* < 0.001) and locations (χ^2^ = 452.21, df = 7, and *p* < 0.001). In Ghana, the prevalence was significantly higher than in Burkina Faso, at 86.38% and 20.76%, respectively (Table 2 and Fig. 3). In Ghana, the prevalence varied significantly with location (χ^2^ = 125.43, df = 5, and *p* < 0.001), with a prevalence of 100% in some locations such as Sissili Bridge, Fumbissi, Kumpole, and Grogro (Fig. 3 and Supplementary Table 3).

The most frequently found trypanosomes were *Tz* and *Tv*, with a prevalence of 30.2% and 22.42%, respectively. However, only *Tz* varied significantly with country (χ^2^ = 7.54, df = 1, and *p* = 0.006) and location (χ^2^ = 185.82, df = 7, and *p* < 0.001). *Trypanosoma congolense* was found in the two locations in Burkina Faso (Comoe at 2.37% and Folonzo at 2.00%), and only in one location in Ghana (Walewale (2.87%)). Its prevalence varied significantly with country (χ^2^ = 6.426, df = 1, and *p* = 0.01) and location (χ^2^ = 34.97, df = 7, and *p* < 0.001).

The *TvTz* multiple infection was the most prevalent in the samples (11.22%). In Ghana, no *TcTv* double infections were found, while in Burkina Faso, no triple infections *TcTvTz* were found. The prevalence of the double infections varied only according with location (χ^2^ = 245.15, df = 7, and *p* < 0.001) (Fig. 3 and *TcTz* Supplementary Table 3).

### Interaction between *Spiroplasma* and *Trypanosoma*

#### Prevalence of co-infections

**Figure 4:**
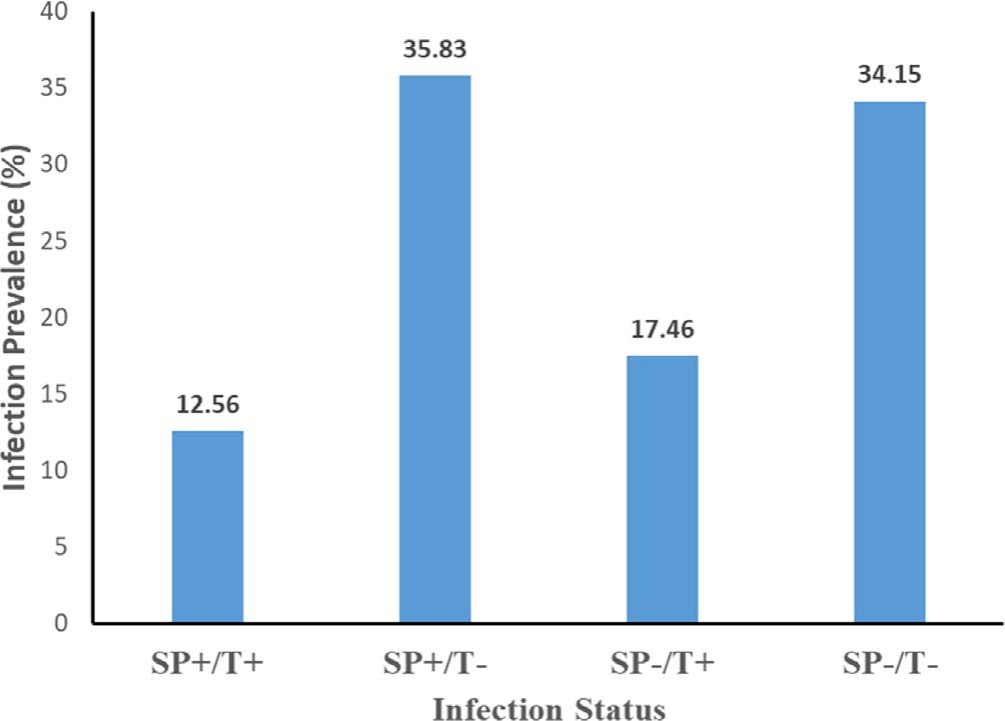
Prevalence of co-infection *Spiroplasma-Trypanosoma* in wild *G. tachinoides*.

The results of the analysis showed that 12.56% of the flies were infected both with *Spiroplasma* and *Trypanosoma*, regardless of country, location, and sex. However, the prevalence of single infections of *Spiroplasma* (35.83%) was higher than that of *Trypanosoma* (17.46%) (Fig. 4). The association between *Spiroplasma* and *Trypanosoma* infections was analyzed using the Cochran-Manthel-Haenzel (CMH) test and chi-square test. Across all samples, the CMH test showed a significant deviation from independence between the two infections (χ^2^MH = 5.19, df = 1, *p* = 0.02). The chi-square test confirmed that the independence between *Spiroplasma* and *Trypanosoma* infections was significant with a Bonferroni correction of *α* = 0.006 (χ^2^ = 9.85, *p* = 0.03). However, when considering countries, only in Ghana the chi-square test did show a significant deviation from independence between the two microbial infections (χ^2^ = 13.004, *p* < 0.001) (Table 3 and Supplementary Table 4).

**Table 3:** Distribution of the association between the presence of *Trypanosoma* spp. and the presence of *Spiroplasma* according to country and location.

Glossina species	Country (Area, Collection Date)	*N*	Sp^+^/T+	Sp^+^/T^−^	Sp^−^/T+	Sp^−^/T^−^	χ^2^	*P*
*G. tachinoides*	Burkina Faso (Comoe)	119	1	28	15	75		
*G. tachinoides*	Burkina Faso (Folonzo)	347	27	161	29	130		
Subtotal		466	28	189	44	205	2.01	0.15
*G. tachinoides*	Ghana (Walewale)	108	24	23	43	18		
*G. tachinoides*	Ghana (Sissili Bridge)	6	3	0	3	0		
*G. tachinoides*	Ghana (Fumbissi)	14	4	0	10	0		
*G. tachinoides*	Ghana (Kumpole)	7	1	0	6	0		
*G. tachinoides*	Ghana (Grogro)	11	3	1	7	0		
*G. tachinoides*	Ghana (Mortani)	41	19	21	1	0		
Subtotal		187	54	45	70	18	13.03	0.0003
Total		840	136	279	184	241	9.85	0.001

#### Co-infection and the density of *Spiroplasma*, *Trypanosoma*, and *Wigglesworthia*

**Figure 5:**
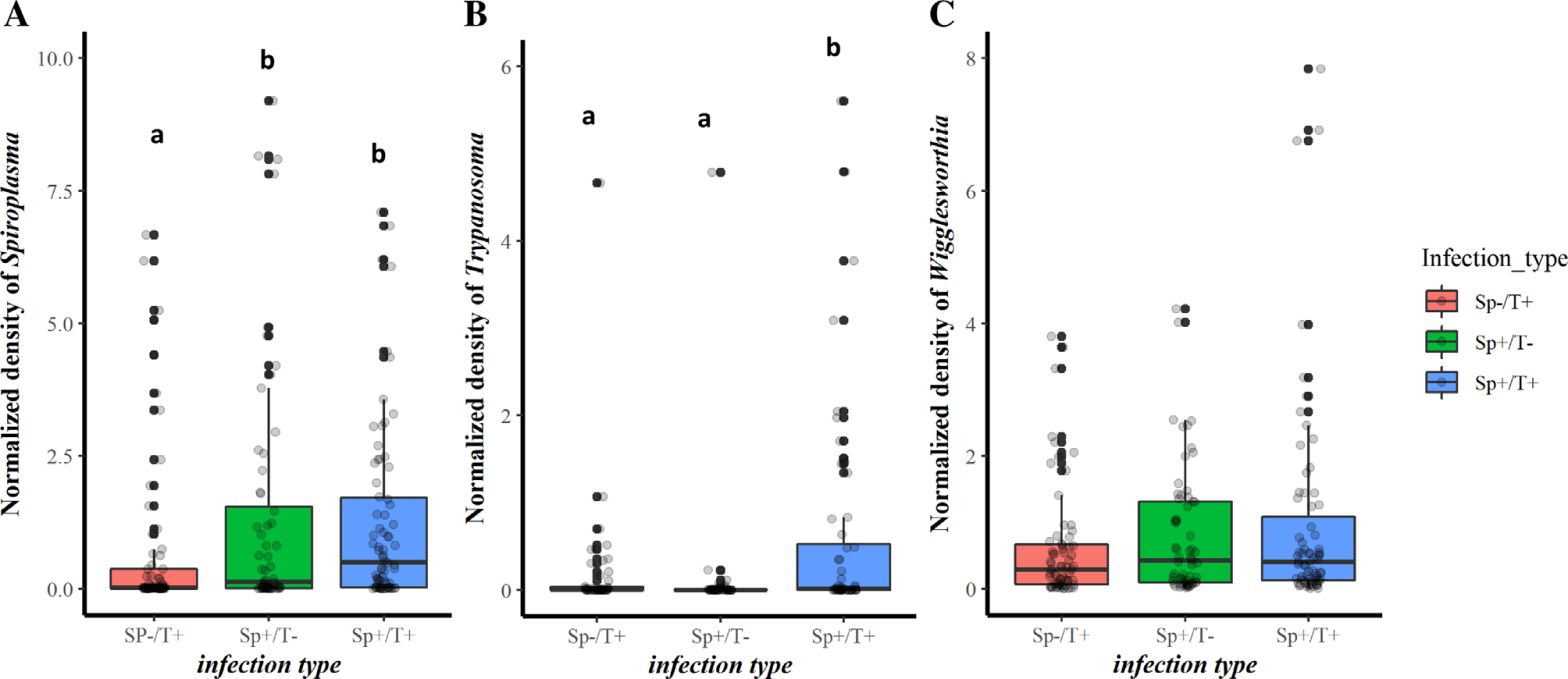
Normalized density of *Spiroplasma* (A), *Trypanosoma* (B), and *Wigglesworthia* (C) according to *Spiroplasma-Trypanosoma* co-infection in wild *G. tachinoides.* Bars marked with the same lower-case letter do not differ significantly at the 0.05 level.

The density of *Spiroplasma*, *Trypanosoma*, and *Wigglesworthia* was evaluated using relative qPCR based on the single (Sp^+^/T^−^; Sp^−^/T^+^) and double co-infection (Sp^+^/T^+^) status. As expected, the results showed that flies infected with *Spiroplasma* (Sp^+^/T^−^ and Sp^+^/T^+^) had a significantly higher density of *Spiroplasma* compared to those not infected (Sp^−^/T^+^), which indicated that flies classified as uninfected by conventional PCR showed lower infection rates with qPCR. However, there was no significant difference in the density of *Spiroplasma* between flies infected with *Spiroplasma* and not infected with *Trypanosoma* (Sp^+^/T^−^) and those infected with both (Sp^+^/T^+^) (Fig. 5A). Furthermore, flies with double co-infection (Sp^+^/T^+^) had a significantly higher density of trypanosomes than those with single co-infection (Sp^+^/T^−^ and Sp^−^/T^+^) (Fig. 5B). However, no significant difference was found in the density of *Wigglesworthia* in the three categories of co-infection (Fig. 5C).

#### Genetic variation and phylogenetic analysis of *Spiroplasma* in wild *G. tachinoides*

**Figure 6:**
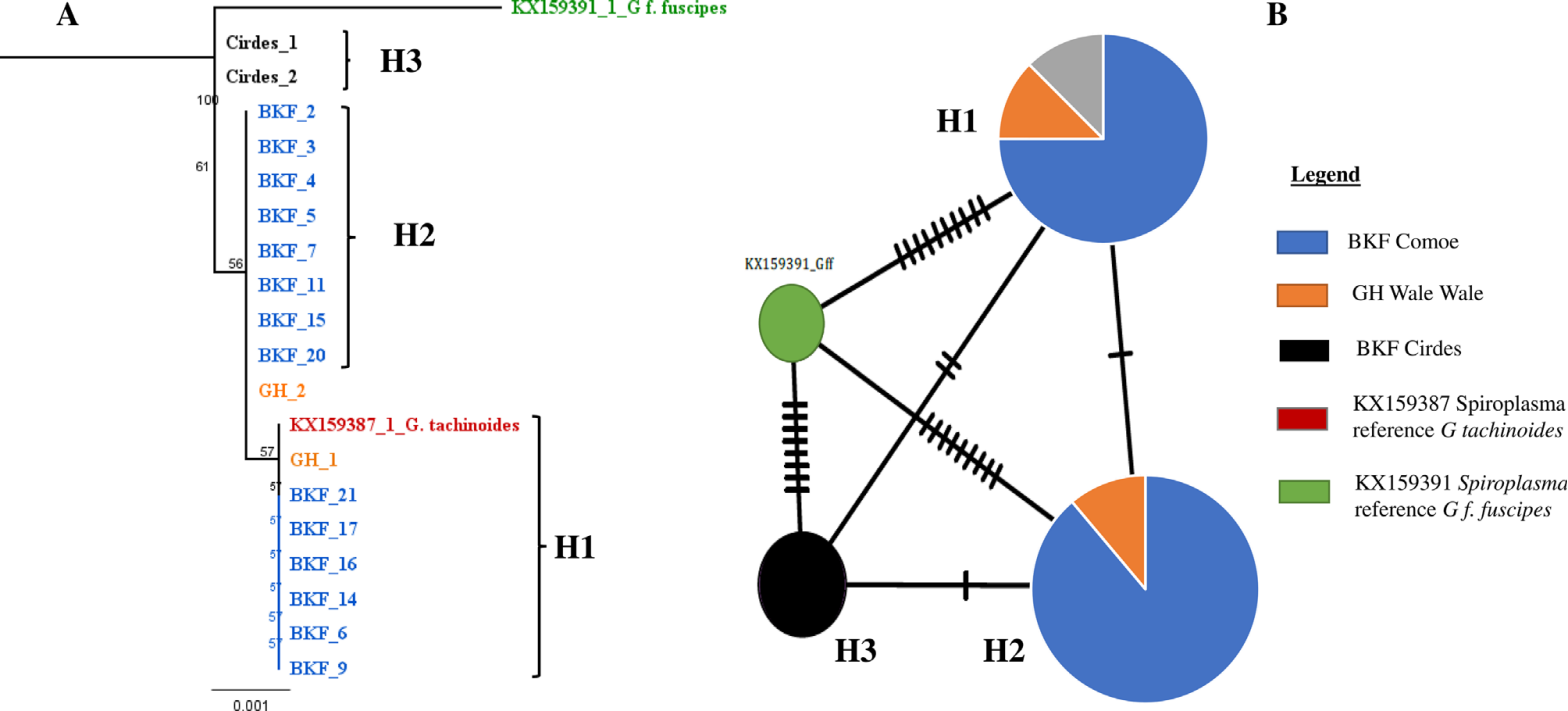
Neighbor-Joining consensus tree (**A**) and Haplotype network analysis (**B**) of the *Spiroplasma* in *G. tachinoides* in Burkina Faso and Ghana. **(A)** Neighbor-Joining consensus tree was built after alignment of all the concatenated sequences. The method used to calculate the distance was Tamura-Nei. **(B)** Haplotype network generated based on the ML tree which was generated based on *Spiroplasma* sequences. The black lineaments on the lines represent mutation events between the haplotypes. The different colors represent the locations. The reference sequence of *Spiroplasma* in *G. fuscipes fuscipes* species (KX159391) was used as the outgroup for construction of both phylogenetic tree and haplotype network.

Among the 35 samples sequenced, 14 sequences from Comoe in Burkina Faso, two from the CIRDES colony, and two from Walewale in Ghana were used for the analysis. For the four genes used for the sequencing, 2,885 base pairs of sequence were generated. The comparison of the sequences showed a global nucleotide mutation rate of 0.06% with two SNPs (Table 4). These two SNPs were found on the *parE* gene (1SNP/745 bp) and *rpoB* gene (1SNP/1455). None of these substitutions were non-synonymous and the percentage of amino-acid mutations was 0.40% (1/248) for the *parE* gene and 0.20% (1/485) for the *rpoB* gene. For the *parE* gene, the mutation resulted in the replacement of isoleucine to valine, but for the *rpoB* gene from phenylalanine to serine. All samples from all locations showed the same profile for the 16S *rRNA* and *fruR* genes. In Burkina Faso and Ghana, two genotypes were found, while only one was detected for CIRDES (Tables 5 and 6). Three different haplotypes were found in the sampling areas with a specific haplotype for the CIRDES colony and Burkina Faso and Ghana sharing the same haplotypes (Table 6, Fig. 6).

**Table 4:** Summary of information on nucleotide polymorphisms detected in the partial sequences of *Spiroplasma* in *G. tachinoides*.

Genes	Length (bp)	No. of SNP/total number of nucleotides (%)	No. of nucleotides substitutions/total no. of nucleotides (%)		No. of amino acid mutations (%)
			No. of silent nucleotide substitutions	No. of non-silent nucleotide substitutions	
16sRNA	352	0/352 (0.00)	–	–	–
*fruR*	333	0/333 (0.00)	–	–	–
*parE*	745	1/745 (0.13)	0/1 (0.00)	1/1 (100)	1/248 (0.40)
*rpoB*	1455	1/1455 (0.06)	0/1 (0.00)	1/1 (100)	1/485 (0.20)
Total	2885	2/2885 (0.06)	0/2 (0.00)	2/2 (100)	2/733 (0.27)

**Table 5:** Alleles of *Spiroplasma* in different locations of tested countries. Numbers between brackets indicate the number of sequences tested per each allele.

Country	Location	No. of flies tested per sequence	16S *rRNA*	*fruR*	*parE*	*rpoB*
Burkina Faso	Comoe	14	1 (14)	1 (14)	1 (6) 2 (8)	1 (14)
	CIRDES*	2	1 (2)	1 (2)	1 (2)	3 (2)
Ghana	Walewale	2	1 (2)	1 (2)	1 (1) 2 (1)	1 (2)

CIRDES*: colony of *G. tachinoides* established in CIRDES insectary.

**Table 6:** *Spiroplasma* haplotypes found in the same individuals collected in East and Southern African countries. The frequency of occurrence of the haplotypes is shown in the last column. The number in parentheses indicates the total number of flies in which the haplotype was detected.

Country	Location	No of flies tested	No of gene profiles	16s RNA	*fruR*	*parE*	*rpoB*	Haplotype No.	Frequency
Burkina Faso	Comoe	14	2	1	1	1	1	H1	6 (14)
				1	1	2	1	H2	8(14)
	CIRDES*	2	1	1	1	1	2	H3	2 (2)
Ghana	Walewale	2	2	1	1	1	1	H1	1 (2)
				1	1	2	1	H2	1 (2)

CIRDES*: colony of *G. tachinoides* established in CIRDES insectary.

## Discussion

In this study, we evaluated the prevalence of the endosymbiont *Spiroplasma* and the *Trypanosoma* parasite in wild *G. tachinoides* in Burkina Faso and Ghana, and the interaction between these two microbes and *Wigglesworthia*. The discovery of the presence of *Spiroplasma* in tsetse flies is quite recent, although its presence in other insects and plants has been known for long time. Doudoumis *et al.* [21] showed the presence of *Spiroplasma* in both laboratory colonies and field populations of *G. tachinoides*, *G f. fuscipes*, and *G. p palpalis*, all belonging to the palpalis group.

The present study confirmed the presence of *Spiroplasma* in *G. tachinoides* in both wild populations and colonized insectary flies. Using 16S *rRNA* gene sequencing, we observed amplification of a bacterial community, different from *Spiroplasma*, in the tsetse species that did not belong to the *palpalis* group. Since the 16S *rRNA* gene is shared with all bacterial species and is one of the most conserved bacterial genes [64], primers designed to target this region could have detected a broad range of bacteria species. It is therefore necessary to carry out taxonomical confirmation by sequencing the respective amplicons [30]. The prevalence in the field was found to be similar to that observed by El Khamlichi *et al.* [23], but higher than that reported by Doudoumis *et al.* [21]. However, the prevalence of *Spiroplasma* in the colony was lower than that observed by Doudoumis *et al.* [21]. The prevalence of the infection varied significantly with location. Furthermore, although the prevalence rates did not differ significantly between Burkina Faso and Ghana, the observed differences in prevalence rates between individual sampling locations suggest that regional variations may impact the infection and the distribution of *Spiroplasma*.

In the study area, three major trypanosomes of humans and animals were found, *T. brucei* s.l. (*Tz*), *T. congolense (Tc)*, and *T. vivax (Tv),* with a relatively high prevalence, particularly in Ghana. This high prevalence of *Trypanosoma* in the flies explains the presence of AAT in the sampling site, highlighting the significant risk of infection in this area. A human infection risk cannot be excluded as *Tz* was identified. Indeed, they are the main cause of HAT [10, 37]. The presence of *Tv* in Burkina Faso was already shown previously [55]. The prevalence of *Trypanosoma* was almost similar to the result of Djohan *et al.* [20] in Côte d’Ivoire (61.4%), but significantly higher (69.97%) than the prevalence obtained by Kame-Ngasse *et al.* [31] in the north of Cameroon (34.81%) and Meharenet and Alemu [38] in Ethiopia. The difference in prevalence compared to the results obtained by Meharenet and Alemu [38] could be due to the diagnostic method used. In their study, the authors used dissection to identify the presence or absence of the parasite, which has some disadvantages, including low sensitivity and susceptibility to the examiner’s technical expertise [12]. Female flies appeared to have higher infection rates than males, which is in line with the results of Meharenet and Alemu [38] and Lefrançois *et al.* [33], and may be due to their longer lifespan. *Trypanozoon sp* and *T. vivax* were the most predominant *Trypanosoma*. Djohan *et al.* [20] also found the same predominant species of trypanosome with *Tv* present at 27.2%. Conversely, Kame-Ngasse *et al.* [31] observed that in *G. tachinoides* in Cameroon, *Tc* was dominant. Since the active foci of the HAT are different, the distribution of parasites will depend on the sampling area. Previous studies have shown the predominance of *Tc* in the “Faro and Deo” region in Cameroon [35, 36].

Mixed infections were predominantly *TvTz* (11.22%) and *TcTz* (4.45%). Previous studies have shown that *G. tachinoides* is commonly infected with various types of trypanosomes. However, the composition of the mixed infections may depend on the distribution of the parasite and the identification method used. For instance, in Côte d’Ivoire, *TvTcs* (9.4%) and *TcTcs* (12.5%) were the predominant mixed infections [20], whereas in Cameroon *TcsTcf* (4.8%) was the predominant one [31].

Our analyses with relative qPCR showed that a *Spiroplasma-Trypanosoma* coinfection had no significant effect on the density of *Wigglesworthia*. This bacterium is an obligate tsetse fly endosymbiont that provides essential nutrients that are absent in blood meals [8]. It is maternally transmitted, making it difficult for other microbiota to invade its niche [3]. Although *Spiroplasma* can be maternally transmitted [56], its presence or absence did not affect the density of *Wigglesworthia* or *Sodalis* in laboratory *G. f. fuscipes* flies.

The analysis of the prevalence of *Spiroplasma* and *Trypanosoma* coinfections suggests a significant deviation from independence, as most of the flies infected with *Spiroplasma* were not infected with *Trypanosoma*, and *vice versa*. This may indicate that the presence of *Spiroplasma* could confer a certain level of refractoriness to *Trypanosoma* infection. This hypothesis was confirmed by the Cochran-Mantel-Haenszel (CMH) and chi-square tests, that showed a significant deviation from independence between the two microorganisms across all samples. Our results align with those of Schneider *et al.* [50] who reported that only 2% of *Spiroplasma* infected flies in *G. f. fuscipes* species were also infected with trypanosomes. The same study also found that, under laboratory conditions, trypanosomes were less likely to colonize the midgut of *G. f. fuscipes* infected with *Spiroplasma*. However, our results did not agree with the higher prevalence of *Spiroplasma* found in *Trypanosoma-*infected *Glossina palpalis palpalis* flies than uninfected ones [40]. The mechanism by which this bacterium enhances refractoriness to trypanosomes in flies remains unclear. It could be related to competition for proliferation niches, given that *Spiroplasma* is found in both the midgut and hemolymph, or to the induction of an immune response in the fly or specific gene regulation. It might also be due to competition for specific nutrients that both microbes need for their development. This has been observed with the endosymbiont *Sodalis*, which competes with the host and parasite for host nutrients [52–54]. Our study indicates that the possible refractory effect of *Spiroplasma* on trypanosome infection is not species-dependent, as it was observed in both *G. tachinoides* and *G. f. fuscipes*. However, these two species belong to the *palpalis* subgroup, within which *Spiroplasma* was exclusively found in our study. Our genotyping showed that the strains of *Spiroplasma* found in *G. tachinoides* in Burkina Faso and Ghana are most closely related to the citri group, as previously reported. This clade is composed of various taxa that are pathogens for plants, such as *S. phoeniceum* [49] and *S*. *citri* [48], as well as protecting Drosophila against nematode infection and parasitic wasps such as *S*. *poulsonii* [29, 44, 65, 66]. Despite belonging to the same citri group, three haplotypes were identified, with Burkina Faso and Ghana sharing two haplotypes and one specific haplotype for the CIRDES colony samples. This information sheds light on the genetic diversity of *Spiroplasma* in the field and in laboratory colonies, which could help us to understand its evolution. The colonization process may induce several mutations that could lead to the development of new haplotypes. Insect microbiota can be influenced by a variety of factors, including environmental conditions, host genetics, and interactions with other organisms. When an insect is colonized, its microbiota may be exposed to different environmental conditions or may interact with new microbial communities, resulting in changes in the composition or function of the microbiota.

The SIT for tsetse flies relies on the release of sterile males within the context of area-wide insect pest management (AW-IPM). To prevent or reduce the transmission of trypanosomes by the released sterile males, they receive at least two blood meals with trypanocidal drugs before being released. However, this is cumbersome and costly, so the discovery that *Spiroplasma* infection could *confer* refractoriness to the trypanosome infection in flies presents an elegant way to mitigate the transmission risk. Releasing sterile males infected with *Spiroplasma*, that are to a certain degree refractory to the trypanosome parasite, would reduce the risk of transmission. Moreover, since paternal transmission of *Spiroplasma* occurs, albeit imperfectly, the offspring from the residual fertility of the sterile males released could also be infected with *Spiroplasma* and be relatively refractory to the parasite. *Spiroplasma* is an endosymbiont that can significantly improve the effectiveness of SIT, making the study and management of this microbe crucial.

## Conclusion

This study reinforced the hypothesis that *Spiroplasma* could enhance refractoriness to trypanosome infections in certain species of tsetse flies, and this would make this symbiont a good candidate for paratransgenesis in addition to *Sodalis*, as previously described. More investigations are required with field samples to better understand interactions between *Spiroplasma* and the trypanosome, and to evaluate the impact of ionizing radiation on the dynamism of *Spiroplasma*.

## Data Availability

Materials described in the paper, including all relevant raw data, are available at this link https://dataverse.harvard.edu/dataset.xhtml?persistentId=doi:10.7910/DVN/ZKIR8I.

## Supplementary materials

The Supplementary materials of this article are available at https://www.parasite-journal.org/10.1051/parasite/2023064/olm. *Supplementary File 1*: R-Marckdown file with details of the data analysis. *Supplementary Table 1*: Details of the geographic coordinates of the sampling sites in Africa. *Supplementary Table 2*: List of Primers used for PCR and quantitative PCR (qPCR) analyses of microbiome in *Glossina tachinoides*. *Supplementary Table 3*: Prevalence in percentage of *Spiroplasma*, *Trypanosoma* spp., and the different *Trypanosoma* species, single or multiple infection in Burkina Faso and Ghana, according to sampling location and sex. Spiro = *Spiroplasma*, T. spp = *Trypanosoma* spp., Tc = *T. congolense*, Tv = *T. vivax*, Tz = *Trypanosoma brucei* spp., TcTv = Coinfection *T. congolense - T. vivax*, TcTz = Coinfection *T. congolense - T. brucei* spp., TvTz = Coinfection *T. vivax - T. brucei* spp., TcTvTZ = Coinfection *T. congolense, T. vivax*, and *T. brucei* spp. Prevalence in percentage of *Spiroplasma*, *Trypanosoma* spp., and the different *Trypanosoma* species, single or multiple infection in Burkina Faso and Ghana, according to sampling location and sex. Spiro = *Spiroplasma*, T. spp. = *Trypanosoma* spp., Tc = *T. congolense*, Tv = *T. vivax*, Tz = *Trypanosoma brucei* spp., TcTv = Coinfection *T. congolense - T. vivax*, TcTz = Coinfection *T. congolense - T. brucei* spp*.*, TvTz = Coinfection *T. vivax - T. brucei* spp*.,* TcTvTZ = Coinfection *T. congolense, T. vivax*, *and T. brucei* spp*.* *Supplementary Table 4*: Chi-2 test of independence between *Spiroplasma* and *Trypanosoma*.
