# Supplementary material for: Prevalence of Spiroplasma and interaction with wild Glossina tachinoides microbiota
Source: Parasite. 2023 Dec 19;30:62. doi: 10.1051/parasite/2023064 (PMC10732139; doi:10.1051/parasite/2023064)
Supplement: Supplementary file 1 — R-Marckdown file with details of the data analysis. Supplementary Table 1: Details of the geographic coordinates of the sampling sites in Africa. Supplementary Table 2: List of Primers used for PCR and quantitative PCR (qPCR) analyses of microbiome in Glossina tachinoides. Supplementary Table 3: Prevalence in percentage of Spiroplasma, Trypanosoma spp., and the different Trypanosoma species, single or multiple infection in Burkina Faso and Ghana, according to sampling location and sex. Spiro = Spiroplasma, T. spp = Trypanosoma spp., Tc = T. congolense, Tv = T. vivax, Tz = Trypanosoma brucei spp., TcTv = Coinfection T. congolense - T. vivax, TcTz = Coinfection T. congolense - T. brucei spp., TvTz = Coinfection T. vivax - T. brucei spp., TcTvTZ = Coinfection T. congolense, T. vivax, and T. brucei spp. Prevalence in percentage of Spiroplasma, Trypanosoma spp., and the different Trypanosoma species, single or multiple infection in Burkina Faso and Ghana, according to sampling location and sex. Spiro = Spiroplasma, T. spp. = Trypanosoma spp., Tc = T. congolense, Tv = T. vivax, Tz = Trypanosoma brucei spp., TcTv = Coinfection T. congolense - T. vivax, TcTz = Coinfection T. congolense - T. brucei spp., TvTz = Coinfection T. vivax - T. brucei spp., TcTvTZ = Coinfection T. congolense, T. vivax, and T. brucei spp. Supplementary Table 4: Chi-2 test of independence between Spiroplasma and Trypanosoma. [file parasite-30-62-s1.zip › parasite230125-1-olm.pdf]

Prevalence of *Spiroplasma* and interaction with wild *Glossina tachinoides* microbiota

Kiswend-sida M. Dera, Mouhamadou M. Dieng, Percy Moyaba, Gisele M. S. Ouedraogo, Soumaïla Pagabeleguem, Chantel J. de Beer, Robert L. Mach, Marc J. B. Vreysen, and Adly M. M. Abd-Alla*

2023-07-28

```
setwd("C:/Users/deram/OneDrive - IAEA/Desktop/Dera/Dera 2/manuscript Spiropla
sma in Gt/manuscipt_02032022/analyse")
#Call libraries
library(ggplot2)
library(lattice)
library(gcookbook)
library(ggfortify)
library(datasets)
library(MASS)
library(survival)
library(rmarkdown)
library(knitr)
library(coxme)

## Loading required package: bdsmatrix

##
## Attaching package: 'bdsmatrix'

## The following object is masked from 'package:base':
##
##     backsolve

library(lme4)

## Loading required package: Matrix

library(nlme)

##
## Attaching package: 'nlme'

## The following object is masked from 'package:lme4':
##
##     lmList

library(tidyverse)

## ── Attaching core tidyverse packages ──────────────────────── tidyverse 2.
0.0 ──
```

```
## ✔ dplyr     1.1.2     ✔ readr     2.1.4
## ✔ forcats   1.0.0     ✔ stringr   1.5.0
## ✔ lubridate 1.9.2     ✔ tibble    3.2.1
## ✔ purrr     1.0.1     ✔ tidyr     1.3.0

## ── Conflicts ────────────────────────────────────────── tidyverse_conflict
s() ──
## ✖ dplyr::collapse() masks nlme::collapse()
## ✖ tidyr::expand()   masks Matrix::expand()
## ✖ dplyr::filter()   masks stats::filter()
## ✖ dplyr::lag()      masks stats::lag()
## ✖ tidyr::pack()     masks Matrix::pack()
## ✖ dplyr::select()   masks MASS::select()
## ✖ tidyr::unpack()   masks Matrix::unpack()
## ℹ Use the conflicted package (<http://conflicted.r-lib.org/>) to force all
conflicts to become errors
```

```
library(gapminder)
library(tufte)
library(dplyr)
library(ggpubr)
library(tufte)
library(ggplot2)
library(lattice)
library(gcookbook)
library(ggfortify)
library(datasets)
library(MASS)
library(survival)
library(rmarkdown)
library(knitr)
library(coxme)
library(lme4)
library(nlme)
library(tufte)
library(ggthemes)
library(AICcmodavg)
```

```
##
## Attaching package: 'AICcmodavg'
##
## The following object is masked from 'package:lme4':
##
##     checkConv
```

```
library(car)
```

```
## Loading required package: carData
##
## Attaching package: 'car'
##
```

```
## The following object is masked from 'package:dplyr':
##
##     recode
##
## The following object is masked from 'package:purrr':
##
##     some
```

Evaluation of the prevalence of Spiroplasma

#loading and preparation of the data

```
spi2 <- read.csv("data_Tryp_Spiro.csv")
str(spi2)

## 'data.frame':    18 obs. of  16 variables:
##  $ Country    : chr  "Burkina Faso" "Burkina Faso" "Burkina Faso" "Burkina Faso" ...
##  $ Location   : chr  "Comoe" "Comoe" "Folonzo" "Folonzo" ...
##  $ Sex        : chr  "F" "M" "F" "M" ...
##  $ Prev_Spiro : num  17.3 29.9 44.6 66.2 57.8 ...
##  $ Prev_Tspp  : num  16.5 15 18.9 32.6 66 ...
##  $ Prev_Tc    : num  1.1 3.64 1.89 2.11 1.89 3.85 0 0 0 0 ...
##  $ Prev_Tv    : num  13.24 11.36 12.26 27.37 9.43 ...
##  $ Prev_Tz    : num  0.37 0 1.89 1.05 24.53 ...
##  $ Prev_Tsg   : int  0 0 0 0 0 0 0 0 0 0 ...
##  $ Prev_TcTv  : num  0.74 0 1.89 0 0 0 0 0 0 0 ...
##  $ Prev_TcTz  : num  1.1 0 0.94 1.05 16.98 ...
##  $ Prev_TcTsg : int  0 0 0 0 0 0 0 0 0 0 ...
##  $ Prev_TvTz  : num  0 0 0 1.05 7.55 14.1 0 0 62.5 0 ...
##  $ Prev_TvTsg : int  0 0 0 0 0 0 0 0 0 0 ...
##  $ Prev_TzTsg : int  0 0 0 0 0 0 0 0 0 0 ...
##  $ Prev_TcTvTz: num  0 0 0 0 5.66 1.28 0 0 0 0 ...

attach(spi2)
head(spi2)

##        Country Location Sex Prev_Spiro Prev_Tspp Prev_Tc Prev_Tv Prev_Tz
## 1 Burkina Faso    Comoe   F      17.31     16.54    1.10   13.24    0.37
## 2 Burkina Faso    Comoe   M      29.85     15.00    3.64   11.36    0.00
## 3 Burkina Faso  Folonzo   F      44.56     18.87    1.89   12.26    1.89
## 4 Burkina Faso  Folonzo   M      66.23     32.63    2.11   27.37    1.05
## 5        Ghana Walewale   F      57.78     66.04    1.89    9.43   24.53
## 6        Ghana Walewale   M      33.33     53.85    3.85   12.82    8.97
##   Prev_Tsg Prev_TcTv Prev_TcTz Prev_TcTsg Prev_TvTz Prev_TvTsg Prev_TzTsg
## 1        0      0.74      1.10          0      0.00          0          0
## 2        0      0.00      0.00          0      0.00          0          0
## 3        0      1.89      0.94          0      0.00          0          0
## 4        0      0.00      1.05          0      1.05          0          0
## 5        0      0.00     16.98          0      7.55          0          0
## 6        0      0.00     10.26          0     14.10          0          0
```

```
##   Prev_TcTvTz
## 1        0.00
## 2        0.00
## 3        0.00
## 4        0.00
## 5        5.66
## 6        1.28
```

```
spi=na.omit(spi2)
summary(spi2)
```

```
##    Country            Location             Sex              Prev_Spiro
##  Length:18          Length:18          Length:18          Min.   :  0.00
##  Class :character   Class :character   Class :character   1st Qu.: 17.98
##  Mode  :character   Mode  :character   Mode  :character   Median : 33.33
##                                                           Mean   : 39.27
##                                                           3rd Qu.: 55.84
##                                                           Max.   :100.00
##
##    Prev_Tspp         Prev_Tc         Prev_Tv          Prev_Tz          Pre
v_Tsg
##  Min.   : 15.00   Min.   :0.000   Min.   : 0.000   Min.   :  0.00   Min.
:0
##  1st Qu.: 45.66   1st Qu.:0.000   1st Qu.: 7.072   1st Qu.:  7.20   1st Qu
.:0
##  Median : 83.33   Median :0.000   Median :13.030   Median : 22.27   Median
:0
##  Mean   : 69.97   Mean   :0.905   Mean   :22.424   Mean   : 30.20   Mean
:0
##  3rd Qu.:100.00   3rd Qu.:1.890   3rd Qu.:30.527   3rd Qu.: 40.62   3rd Qu
.:0
##  Max.   :100.00   Max.   :3.850   Max.   :80.000   Max.   :100.00   Max.
:0
##  NA's   :2        NA's   :2       NA's   :2        NA's   :2        NA's
:2
##    Prev_TcTv        Prev_TcTz        Prev_TcTsg   Prev_TvTz       Prev_TvT
sg
##  Min.   :0.0000   Min.   : 0.000   Min.   :0    Min.   : 0.00   Min.   :0
##  1st Qu.:0.0000   1st Qu.: 0.000   1st Qu.:0    1st Qu.: 0.00   1st Qu.:0
##  Median :0.0000   Median : 0.000   Median :0    Median : 0.00   Median :0
##  Mean   :0.1644   Mean   : 4.456   Mean   :0    Mean   :11.23   Mean   :0
##  3rd Qu.:0.0000   3rd Qu.: 3.390   3rd Qu.:0    3rd Qu.: 8.44   3rd Qu.:0
##  Max.   :1.8900   Max.   :22.220   Max.   :0    Max.   :83.33   Max.   :0
##  NA's   :2        NA's   :2        NA's   :2    NA's   :2       NA's   :2
##    Prev_TzTsg  Prev_TcTvTz
##  Min.   :0    Min.   :0.0000
##  1st Qu.:0    1st Qu.:0.0000
##  Median :0    Median :0.0000
##  Mean   :0    Mean   :0.4338
##  3rd Qu.:0    3rd Qu.:0.0000
```

```
## Max.    :0     Max.    :5.6600
## NA's    :2     NA's    :2

#transform variables to factor
spi2 <- transform(spi2, Country = factor(Country),
                  Location = factor(Location),
                  Sex = factor(Sex))
```

#statistical test to evaluate the significant differences of Spiroplasma between country, location and sex

```
###Best model for the evaluation of Spiroplasma prevalence
model1 <- glm(Prev_Spiro ~ Country, data = spi2, family = gaussian())
model2 <- glm(Prev_Spiro ~ Location, data = spi2, family = gaussian())
model3 <- glm(Prev_Spiro ~ Sex, data = spi2, family = gaussian())
model4 <- glm(Prev_Spiro ~ Country*Location, data = spi2, family = gaussian()
)
model5 <- glm(Prev_Spiro ~ Country+Location, data = spi2, family = gaussian()
)
model6 <- glm(Prev_Spiro ~ Country*Sex, data = spi2, family = gaussian())
model7 <- glm(Prev_Spiro ~ Country+Sex, data = spi2, family = gaussian())
model8 <- glm(Prev_Spiro ~ Location*Sex, data = spi2, family = gaussian())
model9 <- glm(Prev_Spiro ~ Location+Sex, data = spi2, family = gaussian())
model10 <- glm(Prev_Spiro ~ Country+Location+Sex, data = spi2, family = gauss
ian())
model11 <- glm(Prev_Spiro ~ Country*Location*Sex, data = spi2, family = gauss
ian())
model12 <- glm(Prev_Spiro ~ Prev_Tspp, data = spi2, family = gaussian())
model13 <- glm(Prev_Spiro ~ Country + Prev_Tspp, data = spi2, family = gaussi
an())
model14 <- glm(Prev_Spiro ~ Country*Prev_Tspp, data = spi2, family = gaussian
())
model15 <- glm(Prev_Spiro ~ Location + Prev_Tspp, data = spi2, family = gauss
ian())

library(MuMIn)

## Registered S3 methods overwritten by 'MuMIn':
##   method         from
##   formula.coxme coxme
##   logLik.coxme  coxme
##   logLik.lmekin coxme

##
## Attaching package: 'MuMIn'

## The following objects are masked from 'package:AICcmodavg':
##
##     AICc, DIC, importance
```

```
AICc(model1,model2, model3, model4,model5, model6, model7, model8, model9, mo
del10, model11, model12, model13, model14, model15) #mod is the best model

##         df       AICc
## model1   4   180.4038
## model2  10   200.5242
## model3   3   179.3671
## model4  10   200.5242
## model5  10   200.5242
## model6   7   194.0861
## model7   5   184.2612
## model8  19 -1430.7568
## model9  11   214.8927
## model10 11   214.8927
## model11 19 -1423.0102
## model12  3   159.7183
## model13  4   159.1182
## model14  5   159.5679
## model15 10   197.0627

summary(model11)

##
## Call:
## glm(formula = Prev_Spiro ~ Country * Location * Sex, family = gaussian(),
##     data = spi2)
##
## Coefficients: (36 not defined because of singularities)
##                                           Estimate Std. Error t value Pr(>|
t|)
## (Intercept)                                  44.56        NaN     NaN
NaN
## CountryColony                               -30.27        NaN     NaN
NaN
## CountryGhana                                 13.22        NaN     NaN
NaN
## LocationComoe                               -27.25        NaN     NaN
NaN
## LocationFolonzo                                 NA         NA      NA
NA
## LocationFumbissi                            -32.78        NaN     NaN
NaN
## LocationGrogro                              -37.78        NaN     NaN
NaN
## LocationKumpole                             -57.78        NaN     NaN
NaN
## LocationMortani                              42.22        NaN     NaN
NaN
## LocationSissili Bidge                         2.22        NaN     NaN
NaN
```

```
## LocationWalewale                                   NA          NA        NA
NA
## SexM                                            21.67         NaN       NaN
NaN
## CountryColony:LocationComoe                        NA          NA        NA
NA
## CountryGhana:LocationComoe                         NA          NA        NA
NA
## CountryColony:LocationFolonzo                      NA          NA        NA
NA
## CountryGhana:LocationFolonzo                       NA          NA        NA
NA
## CountryColony:LocationFumbissi                     NA          NA        NA
NA
## CountryGhana:LocationFumbissi                      NA          NA        NA
NA
## CountryColony:LocationGrogro                       NA          NA        NA
NA
## CountryGhana:LocationGrogro                        NA          NA        NA
NA
## CountryColony:LocationKumpole                      NA          NA        NA
NA
## CountryGhana:LocationKumpole                       NA          NA        NA
NA
## CountryColony:LocationMortani                      NA          NA        NA
NA
## CountryGhana:LocationMortani                       NA          NA        NA
NA
## CountryColony:LocationSissili Bidge                NA          NA        NA
NA
## CountryGhana:LocationSissili Bidge                 NA          NA        NA
NA
## CountryColony:LocationWalewale                     NA          NA        NA
NA
## CountryGhana:LocationWalewale                      NA          NA        NA
NA
## CountryColony:SexM                             -27.63         NaN       NaN
NaN
## CountryGhana:SexM                              -46.12         NaN       NaN
NaN
## LocationComoe:SexM                              -9.13         NaN       NaN
NaN
## LocationFolonzo:SexM                               NA          NA        NA
NA
## LocationFumbissi:SexM                           32.78         NaN       NaN
NaN
## LocationGrogro:SexM                             54.45         NaN       NaN
NaN
## LocationKumpole:SexM                            74.45         NaN       NaN
NaN
```

```
## LocationMortani:SexM                              21.33        NaN     NaN
NaN
## LocationSissili Bidge:SexM                       -35.55        NaN     NaN
NaN
## LocationWalewale:SexM                                NA         NA      NA
NA
## CountryColony:LocationComoe:SexM                     NA         NA      NA
NA
## CountryGhana:LocationComoe:SexM                      NA         NA      NA
NA
## CountryColony:LocationFolonzo:SexM                   NA         NA      NA
NA
## CountryGhana:LocationFolonzo:SexM                    NA         NA      NA
NA
## CountryColony:LocationFumbissi:SexM                  NA         NA      NA
NA
## CountryGhana:LocationFumbissi:SexM                   NA         NA      NA
NA
## CountryColony:LocationGrogro:SexM                    NA         NA      NA
NA
## CountryGhana:LocationGrogro:SexM                     NA         NA      NA
NA
## CountryColony:LocationKumpole:SexM                   NA         NA      NA
NA
## CountryGhana:LocationKumpole:SexM                    NA         NA      NA
NA
## CountryColony:LocationMortani:SexM                   NA         NA      NA
NA
## CountryGhana:LocationMortani:SexM                    NA         NA      NA
NA
## CountryColony:LocationSissili Bidge:SexM             NA         NA      NA
NA
## CountryGhana:LocationSissili Bidge:SexM              NA         NA      NA
NA
## CountryColony:LocationWalewale:SexM                  NA         NA      NA
NA
## CountryGhana:LocationWalewale:SexM                   NA         NA      NA
NA
##
## (Dispersion parameter for gaussian family taken to be NaN)
##
##     Null deviance: 1.4647e+04  on 17  degrees of freedom
## Residual deviance: 8.7248e-27  on  0  degrees of freedom
## AIC: -1043
##
## Number of Fisher Scoring iterations: 1

summary(model3)
```

```
##
## Call:
## glm(formula = Prev_Spiro ~ Sex, family = gaussian(), data = spi2)
##
## Coefficients:
##             Estimate Std. Error t value Pr(>|t|)
## (Intercept)   37.660     10.069   3.740  0.00178 **
## SexM           3.223     14.240   0.226  0.82379
## ---
## Signif. codes:  0 '***' 0.001 '**' 0.01 '*' 0.05 '.' 0.1 ' ' 1
##
## (Dispersion parameter for gaussian family taken to be 912.4872)
##
##     Null deviance: 14647  on 17  degrees of freedom
## Residual deviance: 14600  on 16  degrees of freedom
## AIC: 177.65
##
## Number of Fisher Scoring iterations: 2
```

```
###Check for model overdispersion with Bolker's function
overdisp_fun <- function(model11) {
  rdf <- df.residual(model11)
  rp <- residuals(model11,type="pearson")
  Pearson.chisq <- sum(rp^2)
  prat <- Pearson.chisq/rdf
  pval <- pchisq(Pearson.chisq, df=rdf, lower.tail=FALSE)
  c(chisq=Pearson.chisq,ratio=prat,rdf=rdf,p=pval)
  }

overdisp_fun(model11)
```

```
##        chisq        ratio          rdf            p
## 8.724802e-27          Inf 0.000000e+00 0.000000e+00
```

```
#difference by country
summary(model1)
```

```
##
## Call:
## glm(formula = Prev_Spiro ~ Country, family = gaussian(), data = spi2)
##
## Coefficients:
##               Estimate Std. Error t value Pr(>|t|)
## (Intercept)     39.487     14.623   2.700   0.0164 *
## CountryColony  -28.177     25.328  -1.113   0.2834
## CountryGhana     4.373     16.885   0.259   0.7992
## ---
## Signif. codes:  0 '***' 0.001 '**' 0.01 '*' 0.05 '.' 0.1 ' ' 1
##
## (Dispersion parameter for gaussian family taken to be 855.3347)
##
```

```
##     Null deviance: 14647  on 17  degrees of freedom
## Residual deviance: 12830  on 15  degrees of freedom
## AIC: 177.33
##
## Number of Fisher Scoring iterations: 2
```

```
anova(model1)
```

```
## Analysis of Deviance Table
##
## Model: gaussian, link: identity
##
## Response: Prev_Spiro
##
## Terms added sequentially (first to last)
##
##
##         Df Deviance Resid. Df Resid. Dev
## NULL                       17      14647
## Country  2   1816.5        15      12830
```

```
Anova(model1)
```

```
## Analysis of Deviance Table (Type II tests)
##
## Response: Prev_Spiro
##         LR Chisq Df Pr(>Chisq)
## Country   2.1238  2     0.3458
```

```
#difference between the Locations
summary(model2)
```

```
##
## Call:
## glm(formula = Prev_Spiro ~ Location, family = gaussian(), data = spi2)
##
## Coefficients:
##                         Estimate Std. Error t value Pr(>|t|)
## (Intercept)                11.31      15.22   0.743  0.47637
## LocationComoe              12.27      21.52   0.570  0.58259
## LocationFolonzo            44.09      21.52   2.048  0.07082 .
## LocationFumbissi           17.86      21.52   0.830  0.42826
## LocationGrogro             23.69      21.52   1.101  0.29963
## LocationKumpole            13.69      21.52   0.636  0.54059
## LocationMortani            87.13      21.52   4.048  0.00289 **
## LocationSissili Bidge      18.69      21.52   0.868  0.40778
## LocationWalewale           34.25      21.52   1.591  0.14607
## ---
## Signif. codes:  0 '***' 0.001 '**' 0.01 '*' 0.05 '.' 0.1 ' ' 1
##
## (Dispersion parameter for gaussian family taken to be 463.2938)
```

```
##
##     Null deviance: 14646.6  on 17  degrees of freedom
## Residual deviance:  4169.6  on  9  degrees of freedom
## AIC: 169.1
##
## Number of Fisher Scoring iterations: 2
```

```
anova(model2)
```

```
## Analysis of Deviance Table
##
## Model: gaussian, link: identity
##
## Response: Prev_Spiro
##
## Terms added sequentially (first to last)
##
##
##          Df Deviance Resid. Df Resid. Dev
## NULL                        17    14646.6
## Location  8    10477         9     4169.6
```

```
Anova(model2)
```

```
## Analysis of Deviance Table (Type II tests)
##
## Response: Prev_Spiro
##          LR Chisq Df Pr(>Chisq)
## Location   22.614  8   0.003897 **
## ---
## Signif. codes:  0 '***' 0.001 '**' 0.01 '*' 0.05 '.' 0.1 ' ' 1
```

```
#difference between the sex
summary(model3)
```

```
##
## Call:
## glm(formula = Prev_Spiro ~ Sex, family = gaussian(), data = spi2)
##
## Coefficients:
##             Estimate Std. Error t value Pr(>|t|)
## (Intercept)   37.660     10.069   3.740  0.00178 **
## SexM           3.223     14.240   0.226  0.82379
## ---
## Signif. codes:  0 '***' 0.001 '**' 0.01 '*' 0.05 '.' 0.1 ' ' 1
##
## (Dispersion parameter for gaussian family taken to be 912.4872)
##
##     Null deviance: 14647  on 17  degrees of freedom
## Residual deviance: 14600  on 16  degrees of freedom
## AIC: 177.65
```

```
##
## Number of Fisher Scoring iterations: 2
```

```
anova(model3)
```

```
## Analysis of Deviance Table
##
## Model: gaussian, link: identity
##
## Response: Prev_Spiro
##
## Terms added sequentially (first to last)
##
##
##      Df Deviance Resid. Df Resid. Dev
## NULL                    17      14647
## Sex   1   46.754        16      14600
```

```
Anova(model3)
```

```
## Analysis of Deviance Table (Type II tests)
##
## Response: Prev_Spiro
##     LR Chisq Df Pr(>Chisq)
## Sex 0.051238  1     0.8209
```

```
# only location is significant, so we will present the plot according to the
location

spi2$Location <- relevel(spi2$Location, ref= "Mortani")
model1<-lm(Prev_Spiro ~ Location, data = spi2, family = gaussian())
```

```
## Warning: In lm.fit(x, y, offset = offset, singular.ok = singular.ok, ...)
:
##  extra argument 'family' will be disregarded
```

```
summary(model1)
```

```
##
## Call:
## lm(formula = Prev_Spiro ~ Location, data = spi2, family = gaussian())
##
## Residuals:
##     Min      1Q  Median      3Q     Max
## -30.000  -9.694   0.000   9.694  30.000
##
## Coefficients:
##                        Estimate Std. Error t value Pr(>|t|)
## (Intercept)               98.44      15.22   6.468 0.000116 ***
## LocationCirdes           -87.13      21.52  -4.048 0.002894 **
## LocationComoe            -74.86      21.52  -3.478 0.006961 **
## LocationFolonzo          -43.05      21.52  -2.000 0.076573 .
```

```
## LocationFumbissi        -69.27      21.52  -3.218 0.010515 *
## LocationGrogro          -63.44      21.52  -2.947 0.016290 *
## LocationKumpole         -73.44      21.52  -3.412 0.007726 **
## LocationSissili Bidge   -68.44      21.52  -3.180 0.011191 *
## LocationWalewale        -52.88      21.52  -2.457 0.036337 *
## ---
## Signif. codes:  0 '***' 0.001 '**' 0.01 '*' 0.05 '.' 0.1 ' ' 1
##
## Residual standard error: 21.52 on 9 degrees of freedom
## Multiple R-squared:  0.7153, Adjusted R-squared:  0.4623
## F-statistic: 2.827 on 8 and 9 DF,  p-value: 0.07137
```

```
spi2$Location <- relevel(spi2$Location, ref= "Folonzo")
model1<-glm(Prev_Spiro ~ Location, data = spi2, family = gaussian())
summary(model1)
```

```
##
## Call:
## glm(formula = Prev_Spiro ~ Location, family = gaussian(), data = spi2)
##
## Coefficients:
##                       Estimate Std. Error t value Pr(>|t|)
## (Intercept)              55.40      15.22   3.640   0.0054 **
## LocationMortani          43.05      21.52   2.000   0.0766 .
## LocationCirdes          -44.09      21.52  -2.048   0.0708 .
## LocationComoe           -31.82      21.52  -1.478   0.1735
## LocationFumbissi        -26.23      21.52  -1.219   0.2540
## LocationGrogro          -20.39      21.52  -0.948   0.3681
## LocationKumpole         -30.39      21.52  -1.412   0.1915
## LocationSissili Bidge   -25.39      21.52  -1.180   0.2683
## LocationWalewale         -9.84      21.52  -0.457   0.6584
## ---
## Signif. codes:  0 '***' 0.001 '**' 0.01 '*' 0.05 '.' 0.1 ' ' 1
##
## (Dispersion parameter for gaussian family taken to be 463.2938)
##
##     Null deviance: 14646.6  on 17  degrees of freedom
## Residual deviance:  4169.6  on  9  degrees of freedom
## AIC: 169.1
##
## Number of Fisher Scoring iterations: 2
```

```
spi2$Location <- relevel(spi2$Location, ref= "Cirdes")
model1<-glm(Prev_Spiro ~ Location, data = spi2, family = gaussian())
summary(model1)
```

```
##
## Call:
## glm(formula = Prev_Spiro ~ Location, family = gaussian(), data = spi2)
##
## Coefficients:
```

```
##                           Estimate Std. Error t value Pr(>|t|)
## (Intercept)                  11.31      15.22   0.743  0.47637
## LocationFolonzo              44.08      21.52   2.048  0.07082 .
## LocationMortani              87.13      21.52   4.048  0.00289 **
## LocationComoe                12.27      21.52   0.570  0.58259
## LocationFumbissi             17.86      21.52   0.830  0.42826
## LocationGrogro               23.69      21.52   1.101  0.29963
## LocationKumpole              13.69      21.52   0.636  0.54059
## LocationSissili Bidge        18.69      21.52   0.868  0.40778
## LocationWalewale             34.25      21.52   1.591  0.14607
## ---
## Signif. codes:  0 '***' 0.001 '**' 0.01 '*' 0.05 '.' 0.1 ' ' 1
##
## (Dispersion parameter for gaussian family taken to be 463.2938)
##
##     Null deviance: 14646.6  on 17  degrees of freedom
## Residual deviance:  4169.6  on  9  degrees of freedom
## AIC: 169.1
##
## Number of Fisher Scoring iterations: 2
```

```
#it"s only on Mortani that there is a significant difference

#Significant difference within the location in each country
#for Ghana
spi2_Gh <- subset(spi2,Country=="Ghana")
spi2_Gh
```

```
##    Country      Location Sex Prev_Spiro Prev_Tspp Prev_Tc Prev_Tv Prev_Tz
## 5    Ghana      Walewale   F      57.78     66.04    1.89    9.43   24.53
## 6    Ghana      Walewale   M      33.33     53.85    3.85   12.82    8.97
## 7    Ghana Sissili Bidge   F      60.00    100.00    0.00   20.00   80.00
## 8    Ghana Sissili Bidge   M       0.00    100.00    0.00    0.00  100.00
## 9    Ghana      Fumbissi   F      25.00    100.00    0.00    0.00   37.50
## 10   Ghana      Fumbissi   M      33.33    100.00    0.00   66.67   33.33
## 11   Ghana       Kumpole   F       0.00    100.00    0.00   40.00   60.00
## 12   Ghana       Kumpole   M      50.00    100.00    0.00   50.00   50.00
## 13   Ghana        Grogro   F      20.00    100.00    0.00   80.00   20.00
## 14   Ghana        Grogro   M      50.00    100.00    0.00    0.00   16.67
## 15   Ghana       Mortani   F     100.00     66.67    0.00    0.00   33.33
## 16   Ghana       Mortani   M      96.88     50.00    0.00   15.63   15.63
##    Prev_Tsg Prev_TcTv Prev_TcTz Prev_TcTsg Prev_TvTz Prev_TvTsg Prev_TzTsg
## 5         0         0     16.98          0      7.55          0          0
## 6         0         0     10.26          0     14.10          0          0
## 7         0         0      0.00          0      0.00          0          0
## 8         0         0      0.00          0      0.00          0          0
## 9         0         0      0.00          0     62.50          0          0
## 10        0         0      0.00          0      0.00          0          0
## 11        0         0      0.00          0      0.00          0          0
## 12        0         0      0.00          0      0.00          0          0
```

```
## 13        0         0      0.00          0      0.00          0           0
## 14        0         0      0.00          0     83.33          0           0
## 15        0         0     22.22          0     11.11          0           0
## 16        0         0     18.75          0      0.00          0           0
##    Prev_TcTvTz
## 5         5.66
## 6         1.28
## 7         0.00
## 8         0.00
## 9         0.00
## 10        0.00
## 11        0.00
## 12        0.00
## 13        0.00
## 14        0.00
## 15        0.00
## 16        0.00
```

```
spi2_Gh$Location<- as.factor(spi2_Gh$Location)
spi2_Gh$Location <-relevel(spi2_Gh$Location, ref = "Mortani" )
model1<-glm(Prev_Spiro ~ Location, data = spi2_Gh, family = gaussian())
summary(model1)
```

```
##
## Call:
## glm(formula = Prev_Spiro ~ Location, family = gaussian(), data = spi2_Gh)
##
## Coefficients:
##                          Estimate Std. Error t value Pr(>|t|)
## (Intercept)                 98.44      17.88   5.504  0.00151 **
## LocationFumbissi           -69.27      25.29  -2.739  0.03379 *
## LocationGrogro             -63.44      25.29  -2.508  0.04602 *
## LocationKumpole            -73.44      25.29  -2.904  0.02721 *
## LocationSissili Bidge      -68.44      25.29  -2.706  0.03530 *
## LocationWalewale           -52.88      25.29  -2.091  0.08150 .
## ---
## Signif. codes:  0 '***' 0.001 '**' 0.01 '*' 0.05 '.' 0.1 ' ' 1
##
## (Dispersion parameter for gaussian family taken to be 639.7438)
##
##     Null deviance: 11486.6  on 11  degrees of freedom
## Residual deviance:  3838.5  on  6  degrees of freedom
## AIC: 117.27
##
## Number of Fisher Scoring iterations: 2
```

```
anova(model1)
```

```
## Analysis of Deviance Table
##
## Model: gaussian, link: identity
```

```
##
## Response: Prev_Spiro
##
## Terms added sequentially (first to last)
##
##
##          Df Deviance Resid. Df Resid. Dev
## NULL                         11    11486.6
## Location  5   7648.2          6     3838.5
```

```
Anova(model1)
```

```
## Analysis of Deviance Table (Type II tests)
##
## Response: Prev_Spiro
##          LR Chisq Df Pr(>Chisq)
## Location   11.955  5    0.03541 *
## ---
## Signif. codes:  0 '***' 0.001 '**' 0.01 '*' 0.05 '.' 0.1 ' ' 1
```

```
#For Burkina Faso
spi2_BKF <- subset(spi2,Country=="Burkina Faso")
spi2_BKF
```

```
##        Country Location Sex Prev_Spiro Prev_Tspp Prev_Tc Prev_Tv Prev_Tz
## 1 Burkina Faso    Comoe   F      17.31     16.54    1.10   13.24    0.37
## 2 Burkina Faso    Comoe   M      29.85     15.00    3.64   11.36    0.00
## 3 Burkina Faso  Folonzo   F      44.56     18.87    1.89   12.26    1.89
## 4 Burkina Faso  Folonzo   M      66.23     32.63    2.11   27.37    1.05
##   Prev_Tsg Prev_TcTv Prev_TcTz Prev_TcTsg Prev_TvTz Prev_TvTsg Prev_TzTsg
## 1        0      0.74      1.10          0      0.00          0          0
## 2        0      0.00      0.00          0      0.00          0          0
## 3        0      1.89      0.94          0      0.00          0          0
## 4        0      0.00      1.05          0      1.05          0          0
##   Prev_TcTvTz
## 1           0
## 2           0
## 3           0
## 4           0
```

```
spi2_BKF$Location<- as.factor(spi2_BKF$Location)
spi2_Gh$Location <-relevel(spi2_Gh$Location, ref = "Comoe" )
model1<-glm(Prev_Spiro ~ Location, data = spi2_BKF, family = gaussian())
summary(model1)
```

```
##
## Call:
## glm(formula = Prev_Spiro ~ Location, family = gaussian(), data = spi2_BKF)
##
## Coefficients:
##                  Estimate Std. Error t value Pr(>|t|)
```

```
## (Intercept)      55.395      8.852   6.258   0.0246 *
## LocationComoe  -31.815     12.518  -2.541   0.1262
## ---
## Signif. codes:  0 '***' 0.001 '**' 0.01 '*' 0.05 '.' 0.1 ' ' 1
##
## (Dispersion parameter for gaussian family taken to be 156.7101)
##
##     Null deviance: 1325.61  on 3  degrees of freedom
## Residual deviance:  313.42  on 2  degrees of freedom
## AIC: 34.797
##
## Number of Fisher Scoring iterations: 2
```

```
anova(model1)
```

```
## Analysis of Deviance Table
##
## Model: gaussian, link: identity
##
## Response: Prev_Spiro
##
## Terms added sequentially (first to last)
##
##
##          Df Deviance Resid. Df Resid. Dev
## NULL                         3    1325.61
## Location  1   1012.2         2     313.42
```

```
Anova(model1)
```

```
## Analysis of Deviance Table (Type II tests)
##
## Response: Prev_Spiro
##          LR Chisq Df Pr(>Chisq)
## Location    6.459  1    0.01104 *
## ---
## Signif. codes:  0 '***' 0.001 '**' 0.01 '*' 0.05 '.' 0.1 ' ' 1
```

#boxplot of the evaluation of Spiroplasma according to the location

```
spi.tiff4<-ggplot(spi2,aes(x=Location ,y=Prev_Spiro, fill = Country)) +
  geom_boxplot() + geom_jitter(width=0.1,alpha=0.2)+ ylim(0, 100)
spi.tiff4
```

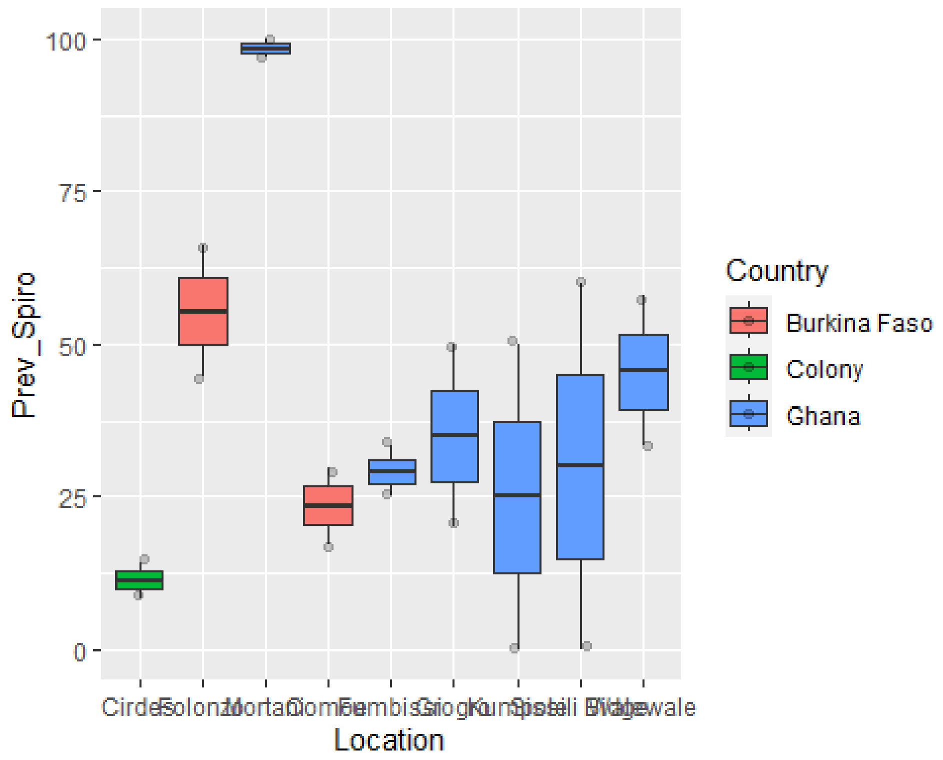


```
tiff("spi.tiff4", width = 7, height = 4, units = 'in', res = 300)
plot(spi.tiff4+theme_tufte() + theme(axis.line = element_line(size = 1, colou
r = "black"))) + xlab(expression(bold("Location"))) + ylab( expression (paste
(bold("Prevalence of "), bolditalic(" Spiroplasma (%)"), )))

## Warning: The `size` argument of `element_line()` is deprecated as of ggplo
t2 3.4.0.
## ℹ Please use the `linewidth` argument instead.
## This warning is displayed once every 8 hours.
## Call `lifecycle::last_lifecycle_warnings()` to see where this warning was
## generated.

## Warning: Removed 1 rows containing missing values (`geom_point()`).

## Warning: Removed 3 rows containing missing values (`geom_point()`).

dev.off()

## png
##   2
```

#Analyse of trypanosoma infection

```
#For Tspp regardless the country
model1<-glm(Prev_Tspp ~ Country, data = spi2)
summary(model1)
```

```
##
## Call:
## glm(formula = Prev_Tspp ~ Country, data = spi2)
##
## Coefficients:
##               Estimate Std. Error t value Pr(>|t|)
## (Intercept)     20.760      9.319   2.228   0.0428 *
## CountryGhana    65.620     10.761   6.098 2.75e-05 ***
## ---
## Signif. codes:  0 '***' 0.001 '**' 0.01 '*' 0.05 '.' 0.1 ' ' 1
##
## (Dispersion parameter for gaussian family taken to be 347.3854)
##
##     Null deviance: 17781.3  on 15  degrees of freedom
## Residual deviance:  4863.4  on 14  degrees of freedom
##   (2 observations deleted due to missingness)
## AIC: 142.88
##
## Number of Fisher Scoring iterations: 2
```

```
anova(model1)
```

```
## Analysis of Deviance Table
##
## Model: gaussian, link: identity
##
## Response: Prev_Tspp
##
## Terms added sequentially (first to last)
##
##
##         Df Deviance Resid. Df Resid. Dev
## NULL                       15    17781.3
## Country  1    12918        14     4863.4
```

```
Anova(model1)
```

```
## Analysis of Deviance Table (Type II tests)
##
## Response: Prev_Tspp
##         LR Chisq Df Pr(>Chisq)
## Country   37.186  1  1.074e-09 ***
## ---
## Signif. codes:  0 '***' 0.001 '**' 0.01 '*' 0.05 '.' 0.1 ' ' 1
```

```
model1<-glm(Prev_Tspp ~ Location, data = spi2)
summary(model1)
```

```
##
## Call:
## glm(formula = Prev_Tspp ~ Location, data = spi2)
```

```
##
## Coefficients:
##                        Estimate Std. Error t value Pr(>|t|)
## (Intercept)              25.750      4.395   5.859 0.000379 ***
## LocationMortani          32.585      6.216   5.242 0.000781 ***
## LocationComoe            -9.980      6.216  -1.606 0.147037
## LocationFumbissi         74.250      6.216  11.945 2.22e-06 ***
## LocationGrogro           74.250      6.216  11.945 2.22e-06 ***
## LocationKumpole          74.250      6.216  11.945 2.22e-06 ***
## LocationSissili Bidge    74.250      6.216  11.945 2.22e-06 ***
## LocationWalewale         34.195      6.216   5.501 0.000573 ***
## ---
## Signif. codes:  0 '***' 0.001 '**' 0.01 '*' 0.05 '.' 0.1 ' ' 1
##
## (Dispersion parameter for gaussian family taken to be 38.63714)
##
##     Null deviance: 17781.3  on 15  degrees of freedom
## Residual deviance:   309.1  on  8  degrees of freedom
##   (2 observations deleted due to missingness)
## AIC: 110.78
##
## Number of Fisher Scoring iterations: 2
```

```
anova(model1)
```

```
## Analysis of Deviance Table
##
## Model: gaussian, link: identity
##
## Response: Prev_Tspp
##
## Terms added sequentially (first to last)
##
##
##          Df Deviance Resid. Df Resid. Dev
## NULL                        15    17781.3
## Location  7    17472         8      309.1
```

```
Anova(model1)
```

```
## Analysis of Deviance Table (Type II tests)
##
## Response: Prev_Tspp
##          LR Chisq Df Pr(>Chisq)
## Location   452.21  7  < 2.2e-16 ***
## ---
## Signif. codes:  0 '***' 0.001 '**' 0.01 '*' 0.05 '.' 0.1 ' ' 1
```

```
model1<-glm(Prev_Tspp ~ Sex, data = spi2)
summary(model1)
```

```
##
## Call:
## glm(formula = Prev_Tspp ~ Sex, data = spi2)
##
## Coefficients:
##             Estimate Std. Error t value Pr(>|t|)
## (Intercept)    71.02      12.59   5.639 6.11e-05 ***
## SexM           -2.08      17.81  -0.117    0.909
## ---
## Signif. codes:  0 '***' 0.001 '**' 0.01 '*' 0.05 '.' 0.1 ' ' 1
##
## (Dispersion parameter for gaussian family taken to be 1268.86)
##
##     Null deviance: 17781  on 15  degrees of freedom
## Residual deviance: 17764  on 14  degrees of freedom
##   (2 observations deleted due to missingness)
## AIC: 163.6
##
## Number of Fisher Scoring iterations: 2
```

```
anova(model1)
```

```
## Analysis of Deviance Table
##
## Model: gaussian, link: identity
##
## Response: Prev_Tspp
##
## Terms added sequentially (first to last)
##
##
##      Df Deviance Resid. Df Resid. Dev
## NULL                    15      17781
## Sex   1   17.306        14      17764
```

```
Anova(model1)
```

```
## Analysis of Deviance Table (Type II tests)
##
## Response: Prev_Tspp
##     LR Chisq Df Pr(>Chisq)
## Sex 0.013639  1      0.907
```

```
#for Tspp in Burkina Faso
spi2_BKF <- subset(spi2,Country=="Burkina Faso")
spi2_BKF
```

```
##        Country Location Sex Prev_Spiro Prev_Tspp Prev_Tc Prev_Tv Prev_Tz
## 1 Burkina Faso    Comoe   F      17.31     16.54    1.10   13.24    0.37
## 2 Burkina Faso    Comoe   M      29.85     15.00    3.64   11.36    0.00
## 3 Burkina Faso  Folonzo   F      44.56     18.87    1.89   12.26    1.89
```

```
## 4 Burkina Faso  Folonzo   M      66.23      32.63     2.11   27.37    1.05
##   Prev_Tsg Prev_TcTv Prev_TcTz Prev_TcTsg Prev_TvTz Prev_TvTsg Prev_TzTsg
## 1        0      0.74      1.10          0      0.00          0          0
## 2        0      0.00      0.00          0      0.00          0          0
## 3        0      1.89      0.94          0      0.00          0          0
## 4        0      0.00      1.05          0      1.05          0          0
##   Prev_TcTvTz
## 1           0
## 2           0
## 3           0
## 4           0

spi2_BKF$Location<- as.factor(spi2_BKF$Location)
spi2_BKF$Location <-relevel(spi2_BKF$Location, ref = "Comoe" )
model1<-glm(Prev_Tspp ~ Location, data = spi2_BKF, family = gaussian())
summary(model1)

##
## Call:
## glm(formula = Prev_Tspp ~ Location, family = gaussian(), data = spi2_BKF)
##
## Coefficients:
##                 Estimate Std. Error t value Pr(>|t|)
## (Intercept)       15.770      4.895   3.221   0.0843 .
## LocationFolonzo    9.980      6.923   1.442   0.2861
## ---
## Signif. codes:  0 '***' 0.001 '**' 0.01 '*' 0.05 '.' 0.1 ' ' 1
##
## (Dispersion parameter for gaussian family taken to be 47.9273)
##
##     Null deviance: 195.455  on 3  degrees of freedom
## Residual deviance:  95.855  on 2  degrees of freedom
## AIC: 30.058
##
## Number of Fisher Scoring iterations: 2

anova(model1)

## Analysis of Deviance Table
##
## Model: gaussian, link: identity
##
## Response: Prev_Tspp
##
## Terms added sequentially (first to last)
##
##
##          Df Deviance Resid. Df Resid. Dev
## NULL                         3    195.455
## Location  1     99.6         2     95.855
```

```
Anova(model1)

## Analysis of Deviance Table (Type II tests)
##
## Response: Prev_Tspp
##          LR Chisq Df Pr(>Chisq)
## Location   2.0782  1     0.1494

#For Tspp in Ghana
spi2_Gh <- subset(spi2,Country=="Ghana")
spi2_Gh

##    Country       Location Sex Prev_Spiro Prev_Tspp Prev_Tc Prev_Tv Prev_Tz
## 5    Ghana       Walewale   F      57.78     66.04    1.89    9.43   24.53
## 6    Ghana       Walewale   M      33.33     53.85    3.85   12.82    8.97
## 7    Ghana Sissili Bidge   F      60.00    100.00    0.00   20.00   80.00
## 8    Ghana Sissili Bidge   M       0.00    100.00    0.00    0.00  100.00
## 9    Ghana       Fumbissi   F      25.00    100.00    0.00    0.00   37.50
## 10   Ghana       Fumbissi   M      33.33    100.00    0.00   66.67   33.33
## 11   Ghana        Kumpole   F       0.00    100.00    0.00   40.00   60.00
## 12   Ghana        Kumpole   M      50.00    100.00    0.00   50.00   50.00
## 13   Ghana         Grogro   F      20.00    100.00    0.00   80.00   20.00
## 14   Ghana         Grogro   M      50.00    100.00    0.00    0.00   16.67
## 15   Ghana        Mortani   F     100.00     66.67    0.00    0.00   33.33
## 16   Ghana        Mortani   M      96.88     50.00    0.00   15.63   15.63
##    Prev_Tsg Prev_TcTv Prev_TcTz Prev_TcTsg Prev_TvTz Prev_TvTsg Prev_TzTsg
## 5         0         0     16.98          0      7.55          0          0
## 6         0         0     10.26          0     14.10          0          0
## 7         0         0      0.00          0      0.00          0          0
## 8         0         0      0.00          0      0.00          0          0
## 9         0         0      0.00          0     62.50          0          0
## 10        0         0      0.00          0      0.00          0          0
## 11        0         0      0.00          0      0.00          0          0
## 12        0         0      0.00          0      0.00          0          0
## 13        0         0      0.00          0      0.00          0          0
## 14        0         0      0.00          0     83.33          0          0
## 15        0         0     22.22          0     11.11          0          0
## 16        0         0     18.75          0      0.00          0          0
##    Prev_TcTvTz
## 5         5.66
## 6         1.28
## 7         0.00
## 8         0.00
## 9         0.00
## 10        0.00
## 11        0.00
## 12        0.00
## 13        0.00
## 14        0.00
```

```
## 15        0.00
## 16        0.00
```

```r
spi2_Gh$Location<- as.factor(spi2_Gh$Location)
spi2_Gh$Location <-relevel(spi2_Gh$Location, ref = "Mortani" )
model1<-glm(Prev_Tspp ~ Location, data = spi2_Gh, family = gaussian())
summary(model1)
```

```
##
## Call:
## glm(formula = Prev_Tspp ~ Location, family = gaussian(), data = spi2_Gh)
##
## Coefficients:
##                          Estimate Std. Error t value Pr(>|t|)
## (Intercept)                58.335      4.215  13.838 8.86e-06 ***
## LocationFumbissi           41.665      5.962   6.989 0.000427 ***
## LocationGrogro             41.665      5.962   6.989 0.000427 ***
## LocationKumpole            41.665      5.962   6.989 0.000427 ***
## LocationSissili Bidge      41.665      5.962   6.989 0.000427 ***
## LocationWalewale            1.610      5.962   0.270 0.796161
## ---
## Signif. codes:  0 '***' 0.001 '**' 0.01 '*' 0.05 '.' 0.1 ' ' 1
##
## (Dispersion parameter for gaussian family taken to be 35.54042)
##
##     Null deviance: 4667.94  on 11  degrees of freedom
## Residual deviance:  213.24  on  6  degrees of freedom
## AIC: 82.585
##
## Number of Fisher Scoring iterations: 2
```

```r
anova(model1)
```

```
## Analysis of Deviance Table
##
## Model: gaussian, link: identity
##
## Response: Prev_Tspp
##
## Terms added sequentially (first to last)
##
##
##          Df Deviance Resid. Df Resid. Dev
## NULL                        11     4667.9
## Location  5   4454.7         6      213.2
```

```r
Anova(model1)
```

```
## Analysis of Deviance Table (Type II tests)
##
## Response: Prev_Tspp
```

```
##          LR Chisq Df Pr(>Chisq)
## Location   125.34  5  < 2.2e-16 ***
## ---
## Signif. codes:  0 '***' 0.001 '**' 0.01 '*' 0.05 '.' 0.1 ' ' 1
```

```
#For Tc
model1<-glm(Prev_Tc ~ Country, data = spi2)
summary(model1)
```

```
##
## Call:
## glm(formula = Prev_Tc ~ Country, data = spi2)
##
## Coefficients:
##                Estimate Std. Error t value Pr(>|t|)
## (Intercept)      2.1850     0.5830   3.748  0.00216 **
## CountryGhana    -1.7067     0.6732  -2.535  0.02380 *
## ---
## Signif. codes:  0 '***' 0.001 '**' 0.01 '*' 0.05 '.' 0.1 ' ' 1
##
## (Dispersion parameter for gaussian family taken to be 1.359705)
##
##     Null deviance: 27.774  on 15  degrees of freedom
## Residual deviance: 19.036  on 14  degrees of freedom
##   (2 observations deleted due to missingness)
## AIC: 54.186
##
## Number of Fisher Scoring iterations: 2
```

```
anova(model1)
```

```
## Analysis of Deviance Table
##
## Model: gaussian, link: identity
##
## Response: Prev_Tc
##
## Terms added sequentially (first to last)
##
##
##         Df Deviance Resid. Df Resid. Dev
## NULL                       15     27.774
## Country  1   8.7381        14     19.036
```

```
Anova(model1)
```

```
## Analysis of Deviance Table (Type II tests)
##
## Response: Prev_Tc
##         LR Chisq Df Pr(>Chisq)
## Country   6.4265  1    0.01124 *
```

```
## ---
## Signif. codes:  0 '***' 0.001 '**' 0.01 '*' 0.05 '.' 0.1 ' ' 1
```

```
model1<-glm(Prev_Tc ~ Location, data = spi2)
summary(model1)
```

```
##
## Call:
## glm(formula = Prev_Tc ~ Location, data = spi2)
##
## Coefficients:
##                         Estimate Std. Error t value Pr(>|t|)
## (Intercept)               2.0000     0.5685   3.518  0.00787 **
## LocationMortani          -2.0000     0.8040  -2.488  0.03766 *
## LocationComoe             0.3700     0.8040   0.460  0.65761
## LocationFumbissi         -2.0000     0.8040  -2.488  0.03766 *
## LocationGrogro           -2.0000     0.8040  -2.488  0.03766 *
## LocationKumpole          -2.0000     0.8040  -2.488  0.03766 *
## LocationSissili Bidge    -2.0000     0.8040  -2.488  0.03766 *
## LocationWalewale          0.8700     0.8040   1.082  0.31072
## ---
## Signif. codes:  0 '***' 0.001 '**' 0.01 '*' 0.05 '.' 0.1 ' ' 1
##
## (Dispersion parameter for gaussian family taken to be 0.64635)
##
##     Null deviance: 27.7740  on 15  degrees of freedom
## Residual deviance:  5.1708  on  8  degrees of freedom
##   (2 observations deleted due to missingness)
## AIC: 45.333
##
## Number of Fisher Scoring iterations: 2
```

```
anova(model1)
```

```
## Analysis of Deviance Table
##
## Model: gaussian, link: identity
##
## Response: Prev_Tc
##
## Terms added sequentially (first to last)
##
##
##          Df Deviance Resid. Df Resid. Dev
## NULL                        15    27.7740
## Location  7   22.603         8     5.1708
```

```
Anova(model1)
```

```
## Analysis of Deviance Table (Type II tests)
##
```

```
## Response: Prev_Tc
##          LR Chisq Df Pr(>Chisq)
## Location   34.971  7  1.133e-05 ***
## ---
## Signif. codes:  0 '***' 0.001 '**' 0.01 '*' 0.05 '.' 0.1 ' ' 1
```

```
model1<-glm(Prev_Tc ~ Sex, data = spi2)
summary(model1)
```

```
##
## Call:
## glm(formula = Prev_Tc ~ Sex, data = spi2)
##
## Coefficients:
##             Estimate Std. Error t value Pr(>|t|)
## (Intercept)   0.6100     0.4853   1.257    0.229
## SexM          0.5900     0.6864   0.860    0.404
##
## (Dispersion parameter for gaussian family taken to be 1.8844)
##
##     Null deviance: 27.774  on 15  degrees of freedom
## Residual deviance: 26.382  on 14  degrees of freedom
##   (2 observations deleted due to missingness)
## AIC: 59.407
##
## Number of Fisher Scoring iterations: 2
```

```
anova(model1)
```

```
## Analysis of Deviance Table
##
## Model: gaussian, link: identity
##
## Response: Prev_Tc
##
## Terms added sequentially (first to last)
##
##
##      Df Deviance Resid. Df Resid. Dev
## NULL                    15     27.774
## Sex   1   1.3924        14     26.382
```

```
Anova(model1)
```

```
## Analysis of Deviance Table (Type II tests)
##
## Response: Prev_Tc
##     LR Chisq Df Pr(>Chisq)
## Sex  0.73891  1       0.39
```

```
#Tv
model1<-glm(Prev_Tv ~ Country, data = spi2)
summary(model1)

##
## Call:
## glm(formula = Prev_Tv ~ Country, data = spi2)
##
## Coefficients:
##              Estimate Std. Error t value Pr(>|t|)
## (Intercept)    16.058     12.525   1.282    0.221
## CountryGhana    8.488     14.463   0.587    0.567
##
## (Dispersion parameter for gaussian family taken to be 627.4919)
##
##     Null deviance: 9001.0  on 15  degrees of freedom
## Residual deviance: 8784.9  on 14  degrees of freedom
##   (2 observations deleted due to missingness)
## AIC: 152.34
##
## Number of Fisher Scoring iterations: 2

anova(model1)

## Analysis of Deviance Table
##
## Model: gaussian, link: identity
##
## Response: Prev_Tv
##
## Terms added sequentially (first to last)
##
##
##         Df Deviance Resid. Df Resid. Dev
## NULL                       15     9001.0
## Country  1   216.16        14     8784.9

Anova(model1)

## Analysis of Deviance Table (Type II tests)
##
## Response: Prev_Tv
##         LR Chisq Df Pr(>Chisq)
## Country  0.34448  1     0.5573

model1<-glm(Prev_Tv ~ Location, data = spi2)
summary(model1)

##
## Call:
## glm(formula = Prev_Tv ~ Location, data = spi2)
```

```
##
## Coefficients:
##                        Estimate Std. Error t value Pr(>|t|)
## (Intercept)              19.815     19.229   1.030    0.333
## LocationMortani         -12.000     27.194  -0.441    0.671
## LocationComoe            -7.515     27.194  -0.276    0.789
## LocationFumbissi         13.520     27.194   0.497    0.632
## LocationGrogro           20.185     27.194   0.742    0.479
## LocationKumpole          25.185     27.194   0.926    0.381
## LocationSissili Bidge    -9.815     27.194  -0.361    0.728
## LocationWalewale         -8.690     27.194  -0.320    0.757
##
## (Dispersion parameter for gaussian family taken to be 739.5328)
##
##     Null deviance: 9001.0  on 15  degrees of freedom
## Residual deviance: 5916.3  on  8  degrees of freedom
##   (2 observations deleted due to missingness)
## AIC: 158.01
##
## Number of Fisher Scoring iterations: 2
```

```
anova(model1)
```

```
## Analysis of Deviance Table
##
## Model: gaussian, link: identity
##
## Response: Prev_Tv
##
## Terms added sequentially (first to last)
##
##
##          Df Deviance Resid. Df Resid. Dev
## NULL                        15     9001.0
## Location  7   3084.8         8     5916.3
```

```
Anova(model1)
```

```
## Analysis of Deviance Table (Type II tests)
##
## Response: Prev_Tv
##          LR Chisq Df Pr(>Chisq)
## Location   4.1713  7     0.7599
```

```
model1<-glm(Prev_Tv ~ Sex, data = spi2)
summary(model1)
```

```
##
## Call:
## glm(formula = Prev_Tv ~ Sex, data = spi2)
##
```

```
## Coefficients:
##             Estimate Std. Error t value Pr(>|t|)
## (Intercept)   21.866      8.962   2.440   0.0286 *
## SexM           1.115     12.675   0.088   0.9311
## ---
## Signif. codes:  0 '***' 0.001 '**' 0.01 '*' 0.05 '.' 0.1 ' ' 1
##
## (Dispersion parameter for gaussian family taken to be 642.5763)
##
##     Null deviance: 9001.0  on 15  degrees of freedom
## Residual deviance: 8996.1  on 14  degrees of freedom
##   (2 observations deleted due to missingness)
## AIC: 152.72
##
## Number of Fisher Scoring iterations: 2
```

```
anova(model1)
```

```
## Analysis of Deviance Table
##
## Model: gaussian, link: identity
##
## Response: Prev_Tv
##
## Terms added sequentially (first to last)
##
##
##      Df Deviance Resid. Df Resid. Dev
## NULL                    15     9001.0
## Sex   1   4.9729        14     8996.1
```

```
Anova(model1)
```

```
## Analysis of Deviance Table (Type II tests)
##
## Response: Prev_Tv
##     LR Chisq Df Pr(>Chisq)
## Sex 0.007739  1     0.9299
```

```
#Tz
model1<-glm(Prev_Tz ~ Country, data = spi2)
summary(model1)
```

```
##
## Call:
## glm(formula = Prev_Tz ~ Country, data = spi2)
##
## Coefficients:
##              Estimate Std. Error t value Pr(>|t|)
## (Intercept)    0.8275    12.3474   0.067   0.9475
## CountryGhana  39.1692    14.2576   2.747   0.0157 *
```

```
## ---
## Signif. codes:  0 '***' 0.001 '**' 0.01 '*' 0.05 '.' 0.1 ' ' 1
##
## (Dispersion parameter for gaussian family taken to be 609.8334)
##
##     Null deviance: 13140.3  on 15  degrees of freedom
## Residual deviance:  8537.7  on 14  degrees of freedom
##   (2 observations deleted due to missingness)
## AIC: 151.88
##
## Number of Fisher Scoring iterations: 2
```

```
anova(model1)
```

```
## Analysis of Deviance Table
##
## Model: gaussian, link: identity
##
## Response: Prev_Tz
##
## Terms added sequentially (first to last)
##
##
##         Df Deviance Resid. Df Resid. Dev
## NULL                       15    13140.3
## Country  1   4602.7        14     8537.7
```

```
Anova(model1)
```

```
## Analysis of Deviance Table (Type II tests)
##
## Response: Prev_Tz
##         LR Chisq Df Pr(>Chisq)
## Country   7.5474  1    0.00601 **
## ---
## Signif. codes:  0 '***' 0.001 '**' 0.01 '*' 0.05 '.' 0.1 ' ' 1
```

```
model1<-glm(Prev_Tz ~ Location, data = spi2)
summary(model1)
```

```
##
## Call:
## glm(formula = Prev_Tz ~ Location, data = spi2)
##
## Coefficients:
##                      Estimate Std. Error t value Pr(>|t|)
## (Intercept)             1.470      5.822   0.252 0.807034
## LocationMortani        23.010      8.234   2.795 0.023392 *
## LocationComoe          -1.285      8.234  -0.156 0.879849
## LocationFumbissi       33.945      8.234   4.123 0.003332 **
## LocationGrogro         16.865      8.234   2.048 0.074709 .
```

```
## LocationKumpole         53.530      8.234   6.501 0.000188 ***
## LocationSissili Bidge   88.530      8.234  10.752 4.93e-06 ***
## LocationWalewale        15.280      8.234   1.856 0.100582
## ---
## Signif. codes:  0 '***' 0.001 '**' 0.01 '*' 0.05 '.' 0.1 ' ' 1
##
## (Dispersion parameter for gaussian family taken to be 67.79524)
##
##     Null deviance: 13140.34  on 15  degrees of freedom
## Residual deviance:   542.36  on  8  degrees of freedom
##   (2 observations deleted due to missingness)
## AIC: 119.78
##
## Number of Fisher Scoring iterations: 2
```

```
anova(model1)
```

```
## Analysis of Deviance Table
##
## Model: gaussian, link: identity
##
## Response: Prev_Tz
##
## Terms added sequentially (first to last)
##
##
##          Df Deviance Resid. Df Resid. Dev
## NULL                        15    13140.3
## Location  7    12598         8      542.4
```

```
Anova(model1)
```

```
## Analysis of Deviance Table (Type II tests)
##
## Response: Prev_Tz
##          LR Chisq Df Pr(>Chisq)
## Location   185.82  7  < 2.2e-16 ***
## ---
## Signif. codes:  0 '***' 0.001 '**' 0.01 '*' 0.05 '.' 0.1 ' ' 1
```

```
model1<-glm(Prev_Tz ~ Sex, data = spi2)
summary(model1)
```

```
##
## Call:
## glm(formula = Prev_Tz ~ Sex, data = spi2)
##
## Coefficients:
##             Estimate Std. Error t value Pr(>|t|)
## (Intercept)   32.202     10.805   2.980  0.00993 **
## SexM          -3.996     15.281  -0.262  0.79750
```

```
## ---
## Signif. codes:  0 '***' 0.001 '**' 0.01 '*' 0.05 '.' 0.1 ' ' 1
##
## (Dispersion parameter for gaussian family taken to be 934.0328)
##
##     Null deviance: 13140  on 15  degrees of freedom
## Residual deviance: 13076  on 14  degrees of freedom
##   (2 observations deleted due to missingness)
## AIC: 158.7
##
## Number of Fisher Scoring iterations: 2
```

```
anova(model1)
```

```
## Analysis of Deviance Table
##
## Model: gaussian, link: identity
##
## Response: Prev_Tz
##
## Terms added sequentially (first to last)
##
##
##      Df Deviance Resid. Df Resid. Dev
## NULL                    15      13140
## Sex   1    63.88        14      13076
```

```
Anova(model1)
```

```
## Analysis of Deviance Table (Type II tests)
##
## Response: Prev_Tz
##     LR Chisq Df Pr(>Chisq)
## Sex 0.068392  1     0.7937
```

```
#TcTv
model1<-glm(Prev_TcTv ~ Country, data = spi2)
summary(model1)
```

```
##
## Call:
## glm(formula = Prev_TcTv ~ Country, data = spi2)
##
## Coefficients:
##                Estimate Std. Error t value Pr(>|t|)
## (Intercept)      0.6575     0.2066   3.182  0.00665 **
## CountryGhana    -0.6575     0.2386  -2.756  0.01546 *
## ---
## Signif. codes:  0 '***' 0.001 '**' 0.01 '*' 0.05 '.' 0.1 ' ' 1
##
## (Dispersion parameter for gaussian family taken to be 0.1707482)
```

```
##
##     Null deviance: 3.6874  on 15  degrees of freedom
## Residual deviance: 2.3905  on 14  degrees of freedom
##   (2 observations deleted due to missingness)
## AIC: 20.988
##
## Number of Fisher Scoring iterations: 2

anova(model1)

## Analysis of Deviance Table
##
## Model: gaussian, link: identity
##
## Response: Prev_TcTv
##
## Terms added sequentially (first to last)
##
##
##         Df Deviance Resid. Df Resid. Dev
## NULL                       15     3.6874
## Country  1   1.2969        14     2.3905

Anova(model1) #p<0.05

## Analysis of Deviance Table (Type II tests)
##
## Response: Prev_TcTv
##         LR Chisq Df Pr(>Chisq)
## Country   7.5955  1   0.005851 **
## ---
## Signif. codes:  0 '***' 0.001 '**' 0.01 '*' 0.05 '.' 0.1 ' ' 1

model1<-glm(Prev_TcTv ~ Location, data = spi2)
summary(model1)

##
## Call:
## glm(formula = Prev_TcTv ~ Location, data = spi2)
##
## Coefficients:
##                         Estimate Std. Error t value Pr(>|t|)
## (Intercept)               0.9450     0.3588   2.634   0.0300 *
## LocationMortani          -0.9450     0.5074  -1.862   0.0996 .
## LocationComoe            -0.5750     0.5074  -1.133   0.2900
## LocationFumbissi         -0.9450     0.5074  -1.862   0.0996 .
## LocationGrogro           -0.9450     0.5074  -1.862   0.0996 .
## LocationKumpole          -0.9450     0.5074  -1.862   0.0996 .
## LocationSissili Bidge    -0.9450     0.5074  -1.862   0.0996 .
## LocationWalewale         -0.9450     0.5074  -1.862   0.0996 .
## ---
```

```
## Signif. codes:  0 '***' 0.001 '**' 0.01 '*' 0.05 '.' 0.1 ' ' 1
##
## (Dispersion parameter for gaussian family taken to be 0.2574812)
##
##     Null deviance: 3.6874  on 15  degrees of freedom
## Residual deviance: 2.0598  on  8  degrees of freedom
##   (2 observations deleted due to missingness)
## AIC: 30.607
##
## Number of Fisher Scoring iterations: 2
```

```
anova(model1)
```

```
## Analysis of Deviance Table
##
## Model: gaussian, link: identity
##
## Response: Prev_TcTv
##
## Terms added sequentially (first to last)
##
##
##          Df Deviance Resid. Df Resid. Dev
## NULL                        15     3.6874
## Location  7   1.6275         8     2.0598
```

```
Anova(model1)
```

```
## Analysis of Deviance Table (Type II tests)
##
## Response: Prev_TcTv
##          LR Chisq Df Pr(>Chisq)
## Location    6.321  7     0.5028
```

```
model1<-glm(Prev_TcTv ~ Sex, data = spi2)
summary(model1)
```

```
##
## Call:
## glm(formula = Prev_TcTv ~ Sex, data = spi2)
##
## Coefficients:
##             Estimate Std. Error t value Pr(>|t|)
## (Intercept)   0.3288     0.1705   1.928   0.0743 .
## SexM         -0.3288     0.2411  -1.364   0.1942
## ---
## Signif. codes:  0 '***' 0.001 '**' 0.01 '*' 0.05 '.' 0.1 ' ' 1
##
## (Dispersion parameter for gaussian family taken to be 0.2325062)
##
##     Null deviance: 3.6874  on 15  degrees of freedom
```

```
## Residual deviance: 3.2551  on 14  degrees of freedom
##   (2 observations deleted due to missingness)
## AIC: 25.928
##
## Number of Fisher Scoring iterations: 2
```

```
anova(model1)
```

```
## Analysis of Deviance Table
##
## Model: gaussian, link: identity
##
## Response: Prev_TcTv
##
## Terms added sequentially (first to last)
##
##
##      Df Deviance Resid. Df Resid. Dev
## NULL                    15     3.6874
## Sex   1  0.43231        14     3.2551
```

```
Anova(model1)
```

```
## Analysis of Deviance Table (Type II tests)
##
## Response: Prev_TcTv
##     LR Chisq Df Pr(>Chisq)
## Sex   1.8593  1     0.1727
```

```
#TcTz
model1<-glm(Prev_TcTz ~ Country, data = spi2)
summary(model1)
```

```
##
## Call:
## glm(formula = Prev_TcTz ~ Country, data = spi2)
##
## Coefficients:
##                Estimate Std. Error t value Pr(>|t|)
## (Intercept)      0.7725     3.9005   0.198    0.846
## CountryGhana     4.9117     4.5039   1.091    0.294
##
## (Dispersion parameter for gaussian family taken to be 60.85507)
##
##     Null deviance: 924.34  on 15  degrees of freedom
## Residual deviance: 851.97  on 14  degrees of freedom
##   (2 observations deleted due to missingness)
## AIC: 115.01
##
## Number of Fisher Scoring iterations: 2
```

```
anova(model1)

## Analysis of Deviance Table
##
## Model: gaussian, link: identity
##
## Response: Prev_TcTz
##
## Terms added sequentially (first to last)
##
##
##         Df Deviance Resid. Df Resid. Dev
## NULL                       15     924.34
## Country  1   72.373        14     851.97

Anova(model1)

## Analysis of Deviance Table (Type II tests)
##
## Response: Prev_TcTz
##         LR Chisq Df Pr(>Chisq)
## Country   1.1893  1     0.2755

model1<-glm(Prev_TcTz ~ Location, data = spi2)
summary(model1)

##
## Call:
## glm(formula = Prev_TcTz ~ Location, data = spi2)
##
## Coefficients:
##                          Estimate Std. Error t value Pr(>|t|)
## (Intercept)                 0.995      1.351   0.736 0.482521
## LocationMortani            19.490      1.911  10.200 7.32e-06 ***
## LocationComoe              -0.445      1.911  -0.233 0.821702
## LocationFumbissi           -0.995      1.911  -0.521 0.616669
## LocationGrogro             -0.995      1.911  -0.521 0.616669
## LocationKumpole            -0.995      1.911  -0.521 0.616669
## LocationSissili Bidge      -0.995      1.911  -0.521 0.616669
## LocationWalewale           12.625      1.911   6.607 0.000168 ***
## ---
## Signif. codes:  0 '***' 0.001 '**' 0.01 '*' 0.05 '.' 0.1 ' ' 1
##
## (Dispersion parameter for gaussian family taken to be 3.651337)
##
##     Null deviance: 924.344  on 15  degrees of freedom
## Residual deviance:  29.211  on  8  degrees of freedom
##   (2 observations deleted due to missingness)
## AIC: 73.037
##
## Number of Fisher Scoring iterations: 2
```

```r
anova(model1)

## Analysis of Deviance Table
##
## Model: gaussian, link: identity
##
## Response: Prev_TcTz
##
## Terms added sequentially (first to last)
##
##
##          Df Deviance Resid. Df Resid. Dev
## NULL                        15     924.34
## Location  7   895.13         8      29.21

Anova(model1) #p<0.05

## Analysis of Deviance Table (Type II tests)
##
## Response: Prev_TcTz
##          LR Chisq Df Pr(>Chisq)
## Location   245.15  7  < 2.2e-16 ***
## ---
## Signif. codes:  0 '***' 0.001 '**' 0.01 '*' 0.05 '.' 0.1 ' ' 1

model1<-glm(Prev_TcTz ~ Sex, data = spi2)
summary(model1)

##
## Call:
## glm(formula = Prev_TcTz ~ Sex, data = spi2)
##
## Coefficients:
##             Estimate Std. Error t value Pr(>|t|)
## (Intercept)    5.155      2.861   1.802   0.0931 .
## SexM          -1.398      4.046  -0.345   0.7349
## ---
## Signif. codes:  0 '***' 0.001 '**' 0.01 '*' 0.05 '.' 0.1 ' ' 1
##
## (Dispersion parameter for gaussian family taken to be 65.4666)
##
##     Null deviance: 924.34  on 15  degrees of freedom
## Residual deviance: 916.53  on 14  degrees of freedom
##   (2 observations deleted due to missingness)
## AIC: 116.17
##
## Number of Fisher Scoring iterations: 2

anova(model1)
```

```
## Analysis of Deviance Table
##
## Model: gaussian, link: identity
##
## Response: Prev_TcTz
##
## Terms added sequentially (first to last)
##
##
##      Df Deviance Resid. Df Resid. Dev
## NULL                     15     924.34
## Sex   1    7.812         14     916.53
```

```
Anova(model1)
```

```
## Analysis of Deviance Table (Type II tests)
##
## Response: Prev_TcTz
##     LR Chisq Df Pr(>Chisq)
## Sex  0.11933  1     0.7298
```

```
#TvTz
model1<-glm(Prev_TvTz ~ Country, data = spi2)
summary(model1)
```

```
##
## Call:
## glm(formula = Prev_TvTz ~ Country, data = spi2)
##
## Coefficients:
##              Estimate Std. Error t value Pr(>|t|)
## (Intercept)    0.2625    12.3725   0.021    0.983
## CountryGhana  14.6200    14.2865   1.023    0.324
##
## (Dispersion parameter for gaussian family taken to be 612.3103)
##
##     Null deviance: 9213.6  on 15  degrees of freedom
## Residual deviance: 8572.3  on 14  degrees of freedom
##   (2 observations deleted due to missingness)
## AIC: 151.95
##
## Number of Fisher Scoring iterations: 2
```

```
anova(model1)
```

```
## Analysis of Deviance Table
##
## Model: gaussian, link: identity
##
## Response: Prev_TvTz
##
```

```
## Terms added sequentially (first to last)
##
##
##         Df Deviance Resid. Df Resid. Dev
## NULL                       15     9213.6
## Country  1   641.23        14     8572.3
```

```
Anova(model1)
```

```
## Analysis of Deviance Table (Type II tests)
##
## Response: Prev_TvTz
##         LR Chisq Df Pr(>Chisq)
## Country   1.0472  1     0.3061
```

```
model1<-glm(Prev_TvTz ~ Location, data = spi2)
summary(model1)
```

```
##
## Call:
## glm(formula = Prev_TvTz ~ Location, data = spi2)
##
## Coefficients:
##                         Estimate Std. Error t value Pr(>|t|)
## (Intercept)                0.525     18.555   0.028    0.978
## LocationMortani            5.030     26.241   0.192    0.853
## LocationComoe             -0.525     26.241  -0.020    0.985
## LocationFumbissi          30.725     26.241   1.171    0.275
## LocationGrogro            41.140     26.241   1.568    0.156
## LocationKumpole           -0.525     26.241  -0.020    0.985
## LocationSissili Bidge     -0.525     26.241  -0.020    0.985
## LocationWalewale          10.300     26.241   0.393    0.705
##
## (Dispersion parameter for gaussian family taken to be 688.5985)
##
##     Null deviance: 9213.6  on 15  degrees of freedom
## Residual deviance: 5508.8  on  8  degrees of freedom
##   (2 observations deleted due to missingness)
## AIC: 156.87
##
## Number of Fisher Scoring iterations: 2
```

```
anova(model1)
```

```
## Analysis of Deviance Table
##
## Model: gaussian, link: identity
##
## Response: Prev_TvTz
##
## Terms added sequentially (first to last)
```

```
##
##
##          Df Deviance Resid. Df Resid. Dev
## NULL                        15     9213.6
## Location  7   3704.8         8     5508.8
```

```
Anova(model1)
```

```
## Analysis of Deviance Table (Type II tests)
##
## Response: Prev_TvTz
##          LR Chisq Df Pr(>Chisq)
## Location   5.3802  7     0.6137
```

```
model1<-glm(Prev_TvTz ~ Sex, data = spi2)
summary(model1)
```

```
##
## Call:
## glm(formula = Prev_TvTz ~ Sex, data = spi2)
##
## Coefficients:
##             Estimate Std. Error t value Pr(>|t|)
## (Intercept)   10.145      9.061   1.120    0.282
## SexM           2.165     12.814   0.169    0.868
##
## (Dispersion parameter for gaussian family taken to be 656.7735)
##
##     Null deviance: 9213.6  on 15  degrees of freedom
## Residual deviance: 9194.8  on 14  degrees of freedom
##   (2 observations deleted due to missingness)
## AIC: 153.07
##
## Number of Fisher Scoring iterations: 2
```

```
anova(model1)
```

```
## Analysis of Deviance Table
##
## Model: gaussian, link: identity
##
## Response: Prev_TvTz
##
## Terms added sequentially (first to last)
##
##
##      Df Deviance Resid. Df Resid. Dev
## NULL                    15     9213.6
## Sex   1   18.749        14     9194.8
```

```
Anova(model1)
```

```
## Analysis of Deviance Table (Type II tests)
##
## Response: Prev_TvTz
##     LR Chisq Df Pr(>Chisq)
## Sex 0.028547  1     0.8658
```

```
#TcTvTz
model1<-glm(Prev_TcTvTz ~ Country, data = spi2)
summary(model1)
```

```
##
## Call:
## glm(formula = Prev_TcTvTz ~ Country, data = spi2)
##
## Coefficients:
##                Estimate Std. Error t value Pr(>|t|)
## (Intercept)  1.665e-16  7.278e-01   0.000    1.000
## CountryGhana 5.783e-01  8.404e-01   0.688    0.503
##
## (Dispersion parameter for gaussian family taken to be 2.118598)
##
##     Null deviance: 30.664  on 15  degrees of freedom
## Residual deviance: 29.660  on 14  degrees of freedom
##   (2 observations deleted due to missingness)
## AIC: 61.282
##
## Number of Fisher Scoring iterations: 2
```

```
anova(model1)
```

```
## Analysis of Deviance Table
##
## Model: gaussian, link: identity
##
## Response: Prev_TcTvTz
##
## Terms added sequentially (first to last)
##
##
##         Df Deviance Resid. Df Resid. Dev
## NULL                       15     30.664
## Country  1   1.0034        14     29.660
```

```
Anova(model1)
```

```
## Analysis of Deviance Table (Type II tests)
##
## Response: Prev_TcTvTz
##         LR Chisq Df Pr(>Chisq)
## Country  0.47362  1     0.4913
```

```r
model1<-glm(Prev_TcTvTz ~ Location, data = spi2)
summary(model1)
```

```
##
## Call:
## glm(formula = Prev_TcTvTz ~ Location, data = spi2)
##
## Coefficients:
##                          Estimate Std. Error t value Pr(>|t|)
## (Intercept)             4.650e-16  7.743e-01   0.000   1.0000
## LocationMortani        -4.095e-16  1.095e+00   0.000   1.0000
## LocationComoe          -1.915e-16  1.095e+00   0.000   1.0000
## LocationFumbissi       -5.175e-16  1.095e+00   0.000   1.0000
## LocationGrogro         -1.976e-15  1.095e+00   0.000   1.0000
## LocationKumpole        -1.813e-16  1.095e+00   0.000   1.0000
## LocationSissili Bidge   0.000e+00  1.095e+00   0.000   1.0000
## LocationWalewale        3.470e+00  1.095e+00   3.169   0.0132 *
## ---
## Signif. codes:  0 '***' 0.001 '**' 0.01 '*' 0.05 '.' 0.1 ' ' 1
##
## (Dispersion parameter for gaussian family taken to be 1.199025)
##
##     Null deviance: 30.6638  on 15  degrees of freedom
## Residual deviance:  9.5922  on  8  degrees of freedom
##   (2 observations deleted due to missingness)
## AIC: 55.22
##
## Number of Fisher Scoring iterations: 2
```

```r
anova(model1)
```

```
## Analysis of Deviance Table
##
## Model: gaussian, link: identity
##
## Response: Prev_TcTvTz
##
## Terms added sequentially (first to last)
##
##
##          Df Deviance Resid. Df Resid. Dev
## NULL                        15    30.6638
## Location  7   21.072         8     9.5922
```

```r
Anova(model1) #p<0.05
```

```
## Analysis of Deviance Table (Type II tests)
##
## Response: Prev_TcTvTz
##          LR Chisq Df Pr(>Chisq)
## Location   17.574  7    0.01405 *
```

```
## ---
## Signif. codes:  0 '***' 0.001 '**' 0.01 '*' 0.05 '.' 0.1 ' ' 1
```

```
model1<-glm(Prev_TcTvTz ~ Sex, data = spi2)
summary(model1)
```

```
##
## Call:
## glm(formula = Prev_TcTvTz ~ Sex, data = spi2)
##
## Coefficients:
##             Estimate Std. Error t value Pr(>|t|)
## (Intercept)   0.7075     0.5129   1.379    0.189
## SexM         -0.5475     0.7254  -0.755    0.463
##
## (Dispersion parameter for gaussian family taken to be 2.104625)
##
##     Null deviance: 30.664  on 15  degrees of freedom
## Residual deviance: 29.465  on 14  degrees of freedom
##   (2 observations deleted due to missingness)
## AIC: 61.176
##
## Number of Fisher Scoring iterations: 2
```

```
anova(model1)
```

```
## Analysis of Deviance Table
##
## Model: gaussian, link: identity
##
## Response: Prev_TcTvTz
##
## Terms added sequentially (first to last)
##
##
##      Df Deviance Resid. Df Resid. Dev
## NULL                    15     30.664
## Sex   1    1.199        14     29.465
```

```
Anova(model1)
```

```
## Analysis of Deviance Table (Type II tests)
##
## Response: Prev_TcTvTz
##     LR Chisq Df Pr(>Chisq)
## Sex  0.56971  1     0.4504
```

##Analyse of the density of Spiroplasma, Trypanosoma and Wigglesworthia according to the co-infection #loading and preparation of the data

```
spi <- read.csv("data_qPCR_Gt_Spiro_Tryp_Wig.csv")
str(spi)

## 'data.frame':    212 obs. of  8 variables:
##  $ Plate            : chr  "P1" "P1" "P1" "P1" ...
##  $ Infection_type   : chr  "SP-/T+" "SP-/T+" "SP-/T+" "SP-/T+" ...
##  $ Species          : chr  "Gt" "Gt" "Gt" "Gt" ...
##  $ Sex              : chr  "M" "M" "M" "M" ...
##  $ Countries        : chr  "GH" "GH" "GH" "GH" ...
##  $ Normalized_Tryp : chr  "0.00018" "0.00005" "N/A" "76.97118" ...
##  $ Normalized_Wig   : num  2.055 1.781 0.337 0.2 0.59 ...
##  $ Normalized_Spiro: chr  "0.243333268" "0.000973628" "0.62409546" "0.0163
91928" ...

attach(spi)

## The following object is masked from spi2:
## 
##     Sex

head(spi)

##   Plate Infection_type Species Sex Countries Normalized_Tryp Normalized_Wi
g
## 1    P1         SP-/T+      Gt   M        GH         0.00018     2.0552980
5
## 2    P1         SP-/T+      Gt   M        GH         0.00005     1.7808980
1
## 3    P1         SP-/T+      Gt   M        GH             N/A     0.3370276
3
## 4    P1         SP-/T+      Gt   M        GH        76.97118     0.1996379
6
## 5    P1         Sp+/T-      Gt   M        GH         0.00011     0.5899923
2
## 6    P1         Sp+/T-      Gt   M        GH         0.00001     0.0533192
4
##   Normalized_Spiro
## 1      0.243333268
## 2      0.000973628
## 3       0.62409546
## 4      0.016391928
## 5      0.002484741
## 6      1.019636868

spi=na.omit(spi)
summary(spi)

##     Plate           Infection_type       Species              Sex           
##  Length:212         Length:212         Length:212         Length:212        
##  Class :character   Class :character   Class :character   Class :character  
##  Mode  :character   Mode  :character   Mode  :character   Mode  :character  
```

```
##
##
##
##   Countries          Normalized_Tryp    Normalized_Wig     Normalized_Spiro
##  Length:212         Length:212         Min.   : 0.00128   Length:212
##  Class :character   Class :character   1st Qu.: 0.10565   Class :character
##  Mode  :character   Mode  :character   Median : 0.40763   Mode  :character
##                                        Mean   : 2.58094
##                                        3rd Qu.: 1.32468
##                                        Max.   :89.95487

# transform into numeric and factor
spi$Normalized_Tryp=as.numeric(spi$Normalized_Tryp)

## Warning: NAs introduced by coercion

spi$Normalized_Wig =as.numeric(spi$Normalized_Wig)
spi$Normalized_Spiro =as.numeric(spi$Normalized_Spiro)

## Warning: NAs introduced by coercion

spi$Infection_type =as.factor(spi$Infection_type)


str(spi)

## 'data.frame':    212 obs. of  8 variables:
##  $ Plate           : chr  "P1" "P1" "P1" "P1" ...
##  $ Infection_type  : Factor w/ 3 levels "SP-/T+","Sp+/T-",..: 1 1 1 1 2 2
2 2 3 3 ...
##  $ Species         : chr  "Gt" "Gt" "Gt" "Gt" ...
##  $ Sex             : chr  "M" "M" "M" "M" ...
##  $ Countries       : chr  "GH" "GH" "GH" "GH" ...
##  $ Normalized_Tryp : num  1.8e-04 5.0e-05 NA 7.7e+01 1.1e-04 ...
##  $ Normalized_Wig  : num  2.055 1.781 0.337 0.2 0.59 ...
##  $ Normalized_Spiro: num  0.243333 0.000974 0.624095 0.016392 0.002485 ...
```

#density of Spiroplasma according to the co-infection

```
spi.tiff1<-ggplot(spi,aes(x=Infection_type ,y=Normalized_Spiro, fill = Infect
ion_type)) +
  geom_boxplot() + geom_jitter(width=0.1,alpha=0.2)+ ylim(0, 10)
spi.tiff1

## Warning: Removed 10 rows containing non-finite values (`stat_boxplot()`).

## Warning: Removed 10 rows containing missing values (`geom_point()`).
```

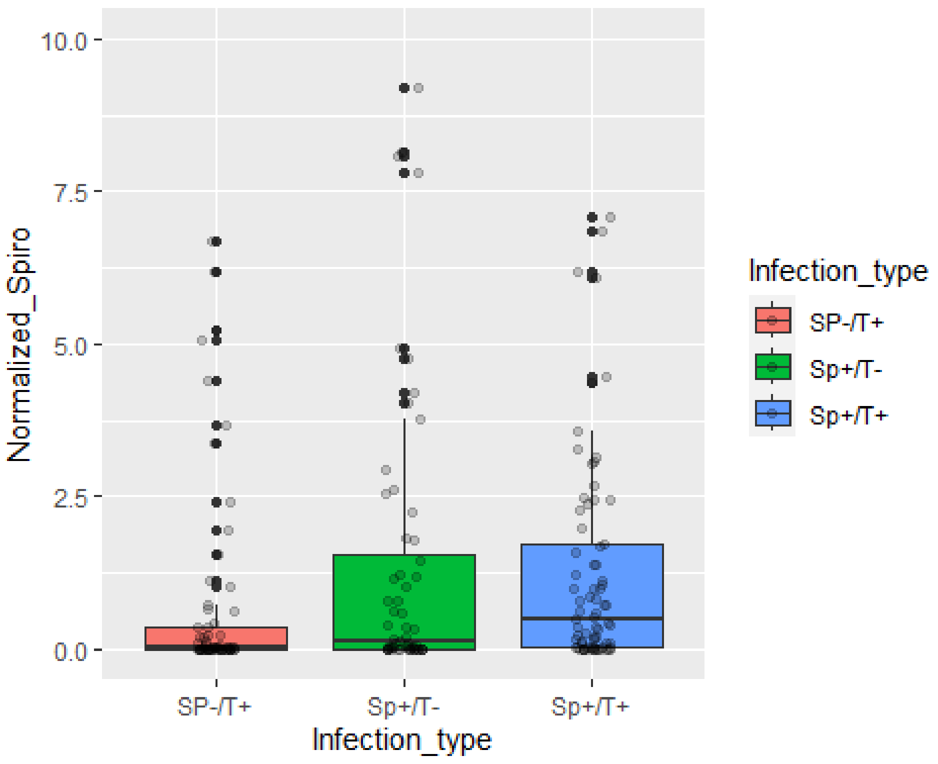


```
model1<-glm(Normalized_Spiro ~ Infection_type, data = spi)
summary(model1)

##
## Call:
## glm(formula = Normalized_Spiro ~ Infection_type, data = spi)
##
## Coefficients:
##                         Estimate Std. Error t value Pr(>|t|)
## (Intercept)                2.999     48.358   0.062    0.951
## Infection_typeSp+/T-      92.848     70.527   1.316    0.189
## Infection_typeSp+/T+      -1.595     67.471  -0.024    0.981
##
## (Dispersion parameter for gaussian family taken to be 166035.1)
##
##     Null deviance: 34589444  on 208  degrees of freedom
## Residual deviance: 34203232  on 206  degrees of freedom
##   (3 observations deleted due to missingness)
## AIC: 3110.3
##
## Number of Fisher Scoring iterations: 2

anova(model1)

## Analysis of Deviance Table
##
## Model: gaussian, link: identity
```

```
##
## Response: Normalized_Spiro
##
## Terms added sequentially (first to last)
##
##
##                 Df Deviance Resid. Df Resid. Dev
## NULL                              208   34589444
## Infection_type  2   386212        206   34203232
```

```
Anova(model1)
```

```
## Analysis of Deviance Table (Type II tests)
##
## Response: Normalized_Spiro
##                 LR Chisq Df Pr(>Chisq)
## Infection_type   2.3261  2     0.3125
```

```
tiff("spi.tiff1", width = 4, height = 4, units = 'in', res = 300)
plot(spi.tiff1+theme_tufte() + theme(axis.line = element_line(size = 1, colou
r = "black"))) + xlab(expression(bolditalic("infection type"))) + ylab( expre
ssion (paste (bold("Normalized density of "), bolditalic(" Spiroplasma"), )))
```

```
## Warning: Removed 10 rows containing non-finite values (`stat_boxplot()`).
## Removed 10 rows containing missing values (`geom_point()`).
```

```
## Warning: Removed 10 rows containing non-finite values (`stat_boxplot()`).
```

```
## Warning: Removed 10 rows containing missing values (`geom_point()`).
```

```
dev.off()
```

```
## png
##   2
```

#density of Wigglesworthia according to the co-infection

```
spi.tiff2<-ggplot(spi,aes(x=Infection_type ,y=Normalized_Wig, fill = Infectio
n_type)) +
  geom_boxplot() + geom_jitter(width=0.1,alpha=0.2)+ ylim(0, 8)
spi.tiff2
```

```
## Warning: Removed 12 rows containing non-finite values (`stat_boxplot()`).
```

```
## Warning: Removed 12 rows containing missing values (`geom_point()`).
```

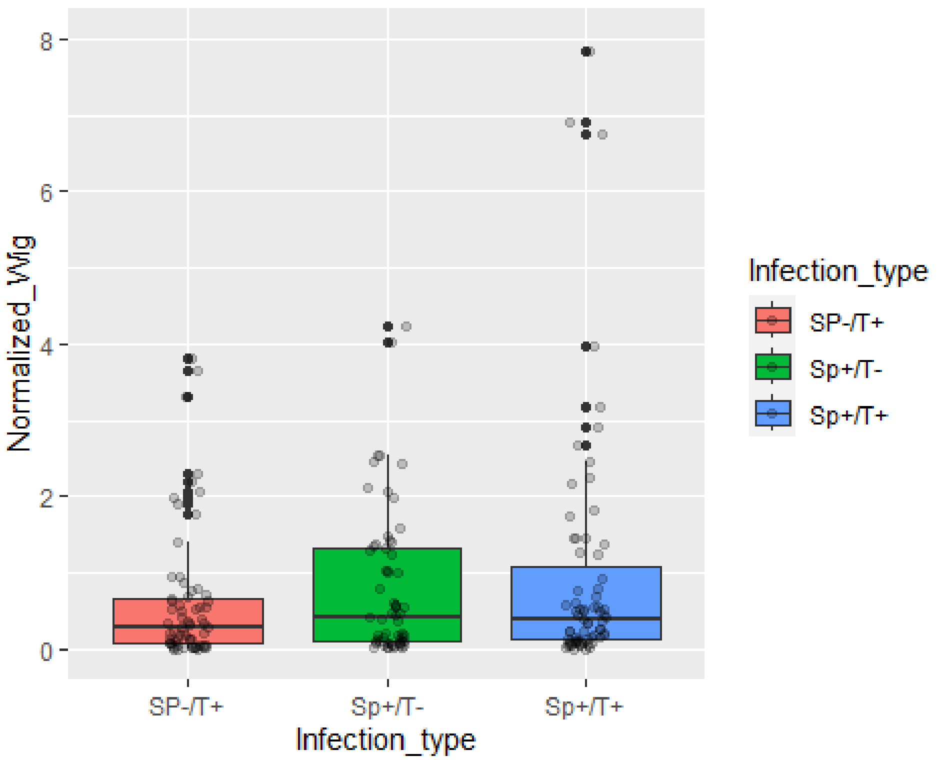


```
model1<-glm(Normalized_Wig ~ Infection_type, data = spi)
summary(model1)

##
## Call:
## glm(formula = Normalized_Wig ~ Infection_type, data = spi)
##
## Coefficients:
##                         Estimate Std. Error t value Pr(>|t|)
## (Intercept)               2.6072     1.1722   2.224   0.0272 *
## Infection_typeSp+/T-    0.1577     1.6955   0.093   0.9260
## Infection_typeSp+/T+   -0.2082     1.6302  -0.128   0.8985
## ---
## Signif. codes:  0 '***' 0.001 '**' 0.01 '*' 0.05 '.' 0.1 ' ' 1
##
## (Dispersion parameter for gaussian family taken to be 97.55097)
##
##     Null deviance: 20393  on 211  degrees of freedom
## Residual deviance: 20388  on 209  degrees of freedom
## AIC: 1577.6
##
## Number of Fisher Scoring iterations: 2

anova(model1)

## Analysis of Deviance Table
##
```

```
## Model: gaussian, link: identity
##
## Response: Normalized_Wig
##
## Terms added sequentially (first to last)
##
##
##                 Df Deviance Resid. Df Resid. Dev
## NULL                              211      20393
## Infection_type  2   4.7645       209      20388
```

```
Anova(model1)
```

```
## Analysis of Deviance Table (Type II tests)
##
## Response: Normalized_Wig
##                 LR Chisq Df Pr(>Chisq)
## Infection_type 0.048841  2     0.9759
```

```
tiff("spi.tiff2", width = 4, height = 4, units = 'in', res = 300)
plot(spi.tiff2+theme_tufte() + theme(axis.line = element_line(size = 1, colou
r = "black"))) + xlab(expression(bolditalic("infection type"))) + ylab( expre
ssion (paste (bold("Normalized density of "), bolditalic(" Wigglesworthia"),
)))
```

```
## Warning: Removed 12 rows containing non-finite values (`stat_boxplot()`).
## Removed 12 rows containing missing values (`geom_point()`).
```

```
## Warning: Removed 12 rows containing non-finite values (`stat_boxplot()`).
```

```
## Warning: Removed 12 rows containing missing values (`geom_point()`).
```

```
dev.off()
```

```
## png
##   2
```

#density of Trypanosoma according to the co-infection

```
spi.tiff3<-ggplot(spi,aes(x=Infection_type ,y=Normalized_Tryp, fill = Infecti
on_type)) +
  geom_boxplot() + geom_jitter(width=0.1,alpha=0.2)+ ylim(0, 6)
spi.tiff3
```

```
## Warning: Removed 54 rows containing non-finite values (`stat_boxplot()`).
```

```
## Warning: Removed 57 rows containing missing values (`geom_point()`).
```

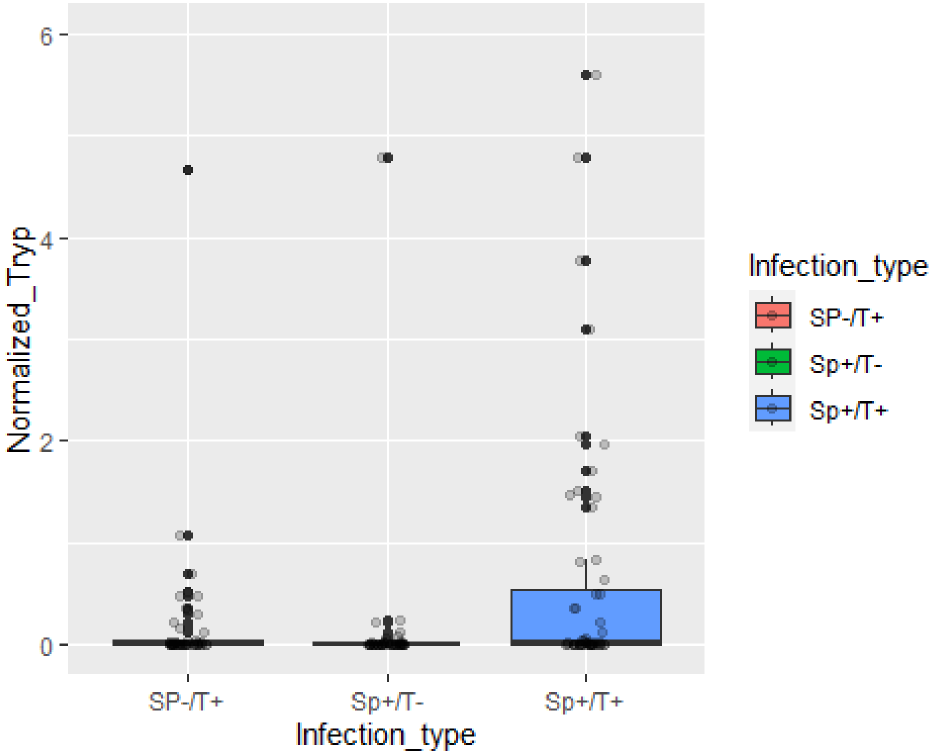


```
model1<-glm(Normalized_Tryp ~ Infection_type, data = spi)
summary(model1)

##
## Call:
## glm(formula = Normalized_Tryp ~ Infection_type, data = spi)
##
## Coefficients:
##                         Estimate Std. Error t value Pr(>|t|)
## (Intercept)                6.301      3.331   1.891   0.0603 .
## Infection_typeSp+/T-      -5.417      4.867  -1.113   0.2673
## Infection_typeSp+/T+       2.234      4.672   0.478   0.6331
## ---
## Signif. codes:  0 '***' 0.001 '**' 0.01 '*' 0.05 '.' 0.1 ' ' 1
##
## (Dispersion parameter for gaussian family taken to be 654.7028)
##
##     Null deviance: 112352  on 171  degrees of freedom
## Residual deviance: 110645  on 169  degrees of freedom
##   (40 observations deleted due to missingness)
## AIC: 1608.4
##
## Number of Fisher Scoring iterations: 2

anova(model1)
```

```
## Analysis of Deviance Table
##
## Model: gaussian, link: identity
##
## Response: Normalized_Tryp
##
## Terms added sequentially (first to last)
##
##
##                Df Deviance Resid. Df Resid. Dev
## NULL                            171     112352
## Infection_type  2   1707.4       169     110645
```

```
Anova(model1)
```

```
## Analysis of Deviance Table (Type II tests)
##
## Response: Normalized_Tryp
##                LR Chisq Df Pr(>Chisq)
## Infection_type   2.6079  2     0.2715
```

```
tiff("spi.tiff3", width = 4, height = 4, units = 'in', res = 300)
plot(spi.tiff3+theme_tufte() + theme(axis.line = element_line(size = 1, colou
r = "black"))) + xlab(expression(bolditalic("infection type"))) + ylab( expre
ssion (paste (bold("Normalized density of "), bolditalic(" Trypanosoma"), )))
```

```
## Warning: Removed 54 rows containing non-finite values (`stat_boxplot()`).
## Removed 57 rows containing missing values (`geom_point()`).
```

```
## Warning: Removed 54 rows containing non-finite values (`stat_boxplot()`).
```

```
## Warning: Removed 57 rows containing missing values (`geom_point()`).
```

```
dev.off()
```

```
## png
##   2
```

#correlation figure between the density of Spiroplasma, Wigglesworthia and Trypanosma

```
reg1a<-ggplot(spi, aes(Normalized_Wig, Normalized_Spiro, col = Infection_type
, fill = Infection_type)) +
  geom_point(size = 3, shape = 21, col = "black") +
  geom_vline(xintercept = 2.1, color = "black", size=1)+
  xlab(expression(italic("Wigglesworthis")))
```

```
## Warning: Using `size` aesthetic for lines was deprecated in ggplot2 3.4.0.
## ℹ Please use `linewidth` instead.
## This warning is displayed once every 8 hours.
## Call `lifecycle::last_lifecycle_warnings()` to see where this warning was
## generated.
```

```
reg1a
## Warning: Removed 3 rows containing missing values (`geom_point()`).
```

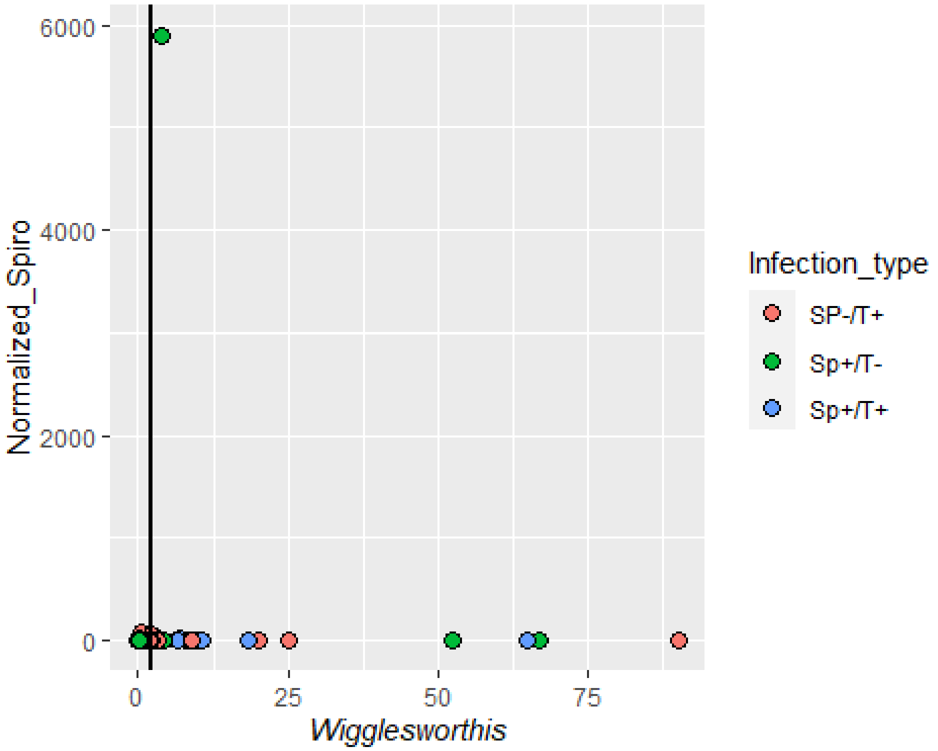


```
tiff("reg1a.tiff", width = 4, height = 4, units = 'in', res = 300)
plot(reg1a+theme_tufte() + theme(axis.line = element_line(size = 1, colour =
"black"))+ theme(legend.position = c(.95, .95),legend.justification = c("righ
t", "top"))) + xlab(expression(bolditalic("Wigglesworthia"))) + ylab(expressi
on(bolditalic("Spiroplasma")))

## Warning: Removed 3 rows containing missing values (`geom_point()`).

## Warning: Removed 3 rows containing missing values (`geom_point()`).

dev.off()

## png
##   2

reg1b<-ggplot(spi, aes(Normalized_Tryp, Normalized_Spiro, col = Infection_typ
e, fill = Infection_type)) +
  geom_point(size = 3, shape = 21, col = "black") +
  geom_vline(xintercept = 2.1, color = "black", size=1)+
  xlab(expression(italic("Wigglesworthis")))
reg1b

## Warning: Removed 41 rows containing missing values (`geom_point()`).
```

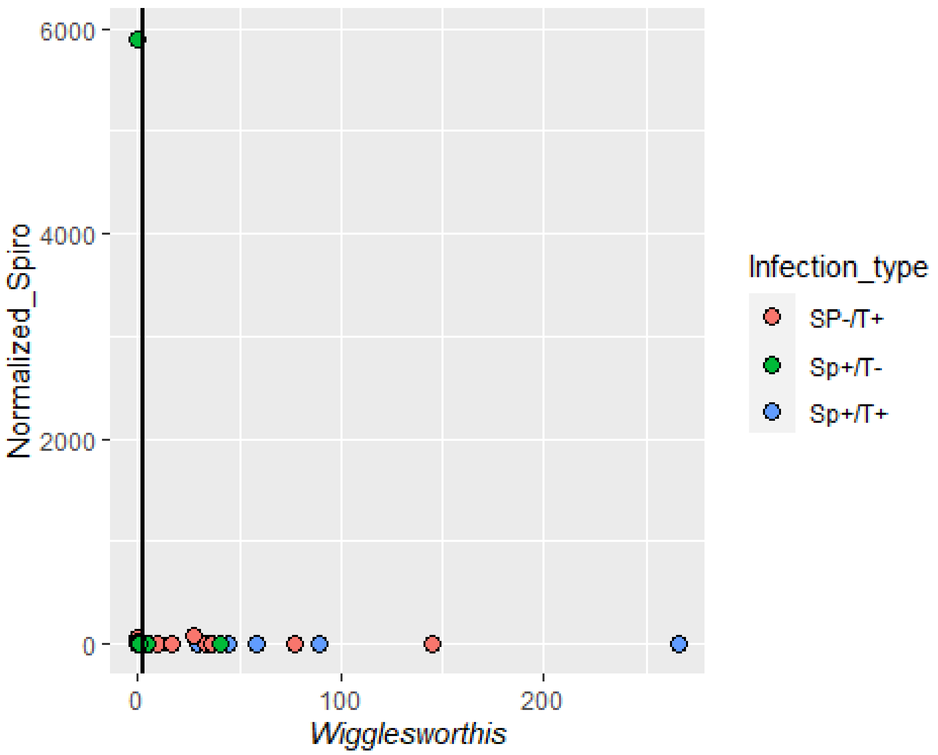


```
tiff("reg1b.tiff", width = 4, height = 4, units = 'in', res = 300)
plot(reg1a+theme_tufte() + theme(axis.line = element_line(size = 1, colour =
"black"))+ theme(legend.position = c(.95, .95),legend.justification = c("righ
t", "top"))) + xlab(expression(bolditalic("Trypanosoma"))) + ylab(expression(
bolditalic("Spiroplasma")))

## Warning: Removed 3 rows containing missing values (`geom_point()`).
## Removed 3 rows containing missing values (`geom_point()`).

dev.off()

## png
##   2
```

Note that the `echo = FALSE` parameter was added to the code chunk to prevent printing of the R code that generated the plot.
