# Supplementary material for: Prevalence of Spiroplasma and interaction with wild Glossina tachinoides microbiota
Source: Parasite. 2023 Dec 19;30:62. doi: 10.1051/parasite/2023064 (PMC10732139; doi:10.1051/parasite/2023064)
Supplement: Supplementary file 1 — R-Marckdown file with details of the data analysis. Supplementary Table 1: Details of the geographic coordinates of the sampling sites in Africa. Supplementary Table 2: List of Primers used for PCR and quantitative PCR (qPCR) analyses of microbiome in Glossina tachinoides. Supplementary Table 3: Prevalence in percentage of Spiroplasma, Trypanosoma spp., and the different Trypanosoma species, single or multiple infection in Burkina Faso and Ghana, according to sampling location and sex. Spiro = Spiroplasma, T. spp = Trypanosoma spp., Tc = T. congolense, Tv = T. vivax, Tz = Trypanosoma brucei spp., TcTv = Coinfection T. congolense - T. vivax, TcTz = Coinfection T. congolense - T. brucei spp., TvTz = Coinfection T. vivax - T. brucei spp., TcTvTZ = Coinfection T. congolense, T. vivax, and T. brucei spp. Prevalence in percentage of Spiroplasma, Trypanosoma spp., and the different Trypanosoma species, single or multiple infection in Burkina Faso and Ghana, according to sampling location and sex. Spiro = Spiroplasma, T. spp. = Trypanosoma spp., Tc = T. congolense, Tv = T. vivax, Tz = Trypanosoma brucei spp., TcTv = Coinfection T. congolense - T. vivax, TcTz = Coinfection T. congolense - T. brucei spp., TvTz = Coinfection T. vivax - T. brucei spp., TcTvTZ = Coinfection T. congolense, T. vivax, and T. brucei spp. Supplementary Table 4: Chi-2 test of independence between Spiroplasma and Trypanosoma. [file parasite-30-62-s1.zip › parasite230125-2-olm.pdf]

<u>Supplementary Table 2:</u> List of Primers used for PCR and quantitative PCR (qPCR) analyses of microbiome in *Glossina tachinoides*

**Target Gene**	**Primer Name**	**Primer Sequence**<br><br>**(Listed 5- to -3)**	**Annealing Temperature (˚C)**	**Amplicon Size (bp)**	**References**
Spiroplasma 16S *rRNA*	63F	GCCTAATACATGCAAGTCGAAC	59 ºC	455	(Doudoumis et al. 2017)
	TKSS	TAGCCGTGGCTTTCTGGTAA			
Spiroplasma fructose repressor (*fruR*)	*fruR-F*	GTCATAATTGCAATTGCTGG	56 ºC /	398	
	FruR-R	CAATGATTAAAGCGGAGGT			
Spiroplasma DNA Topoisomerase 4 subunit B (*parE*)	*ParE-F*	GGAAAATTTGGTGGTGATGG	57 ºC	1126	
	ParE-R	TGGCATTAATCATTACATTAAT TTCT			
RNA polymerase subunit beta (*rpoB*)	*rpoB*	ATGGATCAAACAAATCCATTAG CAGA	60 ºC	1703	
	rpoB	GCATGTAATTTATCATCAACCA TGTGTG			
qPCR *Spiroplasma*	qPCR *Spiroplasma F*	TGAAAAAAACAAACAAATTGT TATTACTTC	56 ◦C	138	
	qPCR *Spiroplasma R*	TTAAGAGCAGTTTCAAAATCAG G			

GpCAG133	GpCAG133-F	ATT TTT GCG TCA ACG TGA	52.5	185-205	(Baker and Krafsur 2001)
	GpCAG133-R	ATG AGG ATG TTG TCC AGT TT			
thiC (thiamine biosynthesis gene)	WiggqPCRthiCF	GACATCAAATCGCGTTACTGG	60	645	(Boucias et al. 2013)
	WiggqPCRthiCR	GACTTGTACGTGATATTTCCAA GC'			
ITS 1	ITS 1-CF	CCG GAA GTT CAC CGA TAT TG	60	250-710	(Njiru et al. 2005)
	ITS 1-BR	TTG CTG CGT TCT TCA ACG AA			
18S *rRNA*	18S_Typ_F	CGC CAA GCT AAT ACA TGA ACC AA	60		Kindly provide by Jan Van Den Abbeele
	18S_Tryp_R	TAA TTT CAT TCA TTC GCT GGA CG			

References

Baker, M. D., and E. S. Krafsur. 2001. 'Identification and Properties of Microsatellite Markers in Tsetse Flies Glossina Morsitans Sensu Lato (Diptera: Glossinidae)'. *Molecular Ecology Notes* 1 (4): 234–36.

Boucias, D. G., H. M. Kariithi, K. Bourtzis, D. I. Schneider, K. Kelley, W. J. Miller, A. G. Parker, and A. M. M. Abd-Alla. 2013. 'Transgenerational Transmission of the *Glossina Pallidipes* Hytrosavirus Depends on the Presence of a Functional Symbiome'. *PLoS One* 8: e61150-.

Doudoumis, V., F. Blow, A. Saridaki, A. Augustinos, N. A. Dyer, I. Goodhead, P. Solano, et al. 2017. 'Challenging the Wigglesworthia, Sodalis, Wolbachia Symbiosis Dogma in Tsetse Flies: Spiroplasma Is Present in Both Laboratory and Natural Populations'. *Scientific Reports* 7 (1). https://doi.org/10.1038/s41598-017-04740-3.

Njiru, Z. K., C. C. Constantine, S. Guya, J. Crowther, J. M. Kiragu, R. C. A. Thompson, and A. M. R. Davila. 2005. 'The Use of ITS1 RDNA PCR in Detecting Pathogenic African Trypanosomes'. *Parasitology* 95: 186–92.
