# Supplementary material for: Prevalence of Spiroplasma and interaction with wild Glossina tachinoides microbiota
Source: Parasite. 2023 Dec 19;30:62. doi: 10.1051/parasite/2023064 (PMC10732139; doi:10.1051/parasite/2023064)
Supplement: Supplementary file 1 — R-Marckdown file with details of the data analysis. Supplementary Table 1: Details of the geographic coordinates of the sampling sites in Africa. Supplementary Table 2: List of Primers used for PCR and quantitative PCR (qPCR) analyses of microbiome in Glossina tachinoides. Supplementary Table 3: Prevalence in percentage of Spiroplasma, Trypanosoma spp., and the different Trypanosoma species, single or multiple infection in Burkina Faso and Ghana, according to sampling location and sex. Spiro = Spiroplasma, T. spp = Trypanosoma spp., Tc = T. congolense, Tv = T. vivax, Tz = Trypanosoma brucei spp., TcTv = Coinfection T. congolense - T. vivax, TcTz = Coinfection T. congolense - T. brucei spp., TvTz = Coinfection T. vivax - T. brucei spp., TcTvTZ = Coinfection T. congolense, T. vivax, and T. brucei spp. Prevalence in percentage of Spiroplasma, Trypanosoma spp., and the different Trypanosoma species, single or multiple infection in Burkina Faso and Ghana, according to sampling location and sex. Spiro = Spiroplasma, T. spp. = Trypanosoma spp., Tc = T. congolense, Tv = T. vivax, Tz = Trypanosoma brucei spp., TcTv = Coinfection T. congolense - T. vivax, TcTz = Coinfection T. congolense - T. brucei spp., TvTz = Coinfection T. vivax - T. brucei spp., TcTvTZ = Coinfection T. congolense, T. vivax, and T. brucei spp. Supplementary Table 4: Chi-2 test of independence between Spiroplasma and Trypanosoma. [file parasite-30-62-s1.zip › parasite230125-3-olm.pdf]

Supplementary table 3: Prevalence in percentage of *Spiroplasma*, *Trypanosoma spp* and the different *Trypanosoma* species, single or multiple infection in Burkina Faso and Ghana, according to the sampling location and the sex. Spiro = *Spiroplasma*, T. spp = *Trypanosoma spp*, Tc = *T. congolense*, Tv = *T. vivax*, Tz = *Trypanosoma brucei spp*, TcTv = Coinfection *T. congolense T. vivax*, TcTz = Coinfection *T. congolense T. brucei spp*, TvTz = Coinfection *T. vivax T. brucei spp*, TcTvTZ = Coinfection *T. congolense, T. vivax and T. brucei spp*

Country	Location	Sex	Spiro	T. spp	Tc	Tv	Tz	TcTv	TcTz	TvTz	TcTvTz
Burkina Faso	Comoe	F	17.31	16.54	1.1	13.24	0.37	0.74	1.1	0	0
Burkina Faso	Comoe	M	29.85	15	3.64	11.36	0	0	0	0	0
Burkina Faso	Folonzo	F	44.56	18.87	1.89	12.26	1.89	1.89	0.94	0	0
Burkina Faso	Folonzo	M	66.23	32.63	2.11	27.37	1.05	0	1.05	1.05	0
Ghana	Walewale	F	57.78	66.04	1.89	9.43	24.53	0	16.98	7.55	5.66
Ghana	Walewale	M	33.33	53.85	3.85	12.82	8.97	0	10.26	14.1	1.28
Ghana	Sissili Bidge	F	60	100	0	20	80	0	0	0	0
Ghana	Sissili Bidge	M	0	100	0	0	100	0	0	0	0
Ghana	Fumbissi	F	25	100	0	0	37.5	0	0	62.5	0
Ghana	Fumbissi	M	33.33	100	0	66.67	33.33	0	0	0	0
Ghana	Kumpole	F	0	100	0	40	60	0	0	0	0
Ghana	Kumpole	M	50	100	0	50	50	0	0	0	0
Ghana	Grogro	F	20	100	0	80	20	0	0	0	0
Ghana	Grogro	M	50	100	0	0	16.67	0	0	83.33	0
Ghana	Mortani	F	100	66.67	0	0	33.33	0	22.22	11.11	0
Ghana	Mortani	M	96.88	50	0	15.63	15.63	0	18.75	0	0
